# Systematic review and network meta-analysis of optimal acupuncture modalities for post-stroke upper limb motor dysfunction

**DOI:** 10.3389/fneur.2026.1770511

**Published:** 2026-04-20

**Authors:** Hanyi Ye, Sulin Tang, Yijun Li

**Affiliations:** Department of Acupuncture, The Affiliated Yangming Hospital of Ningbo University, Ningbo, Zhejiang, China

**Keywords:** acupuncture, NMA, stroke, systematic review, ULMD

## Abstract

**Purpose:**

This study seeks to assess optimal acupuncture modalities in improving motor function (MF), activities of daily living (ADL), spasticity, and other sequelae.

**Methods:**

As of August 29, 2024, we searched eight databases (PubMed, EMBASE, Cochrane Library, Web of Science, China National Knowledge Infrastructure, SinoMed, China Science and Technology Journal Database, Wanfang). The searched reports were screened, and related data were extracted. Study quality was assessed via Cochrane RoB 2.0. Data analyses were performed using R 4.4.2 and Stata 15.

**Results:**

This analysis included 103 studies (9,351 patients). Our analysis indicated that conventional physical therapy (CP) plus body acupuncture (BA) plus electroacupuncture (EA) (CP_BA_EA) (SUCRA = 79.67%) was the most effective for improving MF with treatment > one month. For treatment duration ≤ one month, traditional Chinese medicine (CM) combined with a new minimally invasive acupuncture method (NM) (CM_NM) (SUCRA = 99.96%) was more beneficial. In enhancing ADL, for treatment duration > one month, CP_BA_EA (SUCRA = 99.13%) was superior; (CP_SA_BA(SUCRA=96.43%) was optimal for treatment duration ≤ one month. Regarding upper limb hypertonia, CP plus scalp acupuncture (SA) plus BA (CP_SA_BA) was most effective when the treatment duration exceeded one month (SUCRA = 81.53%); for the treatment duration ≤ one month, CP_SA_BA was optimal (SUCRA = 87.77%).

**Conclusion:**

No monotherapy comprehensively relieves upper limb motor dysfunction, while combination therapies appear to be promising. Optimal interventions vary by treatment duration even for identical outcomes. However, in light of the cautious stance adopted by major international guidelines and the inherent methodological limitations, acupuncture should be regarded as a potential adjunctive intervention rather than an independent therapy.

**Systematic review registration:**

https://www.crd.york.ac.uk/PROSPERO/view/CRD42024589860 in PROSPERO, CRD42024589860.

## 1 Introduction

Stroke is a localized brain dysfunction caused by acute cerebrovascular events and clinically categorized into hemorrhagic and ischemic stroke. Upper limb motor dysfunction (ULMD) affects approximately 55–75% of post-stroke patients (1), with typical manifestations including motor impairment, sensory deficits, and higher-order cognitive dysfunction. Evidence indicates that only 38% of patients regain partial dexterity in the affected upper limb six months after stroke, 15% recover approximately half of their hand function, and merely 3% achieve greater than 70% restoration of baseline hand function (2). These findings highlight that ULMD remains a critical challenge in stroke rehabilitation. Current rehabilitation strategies for stroke can be broadly categorized into Western medicine (WM)-based and traditional Chinese medicine (CM)-based approaches. Western rehabilitation includes occupational therapy, functional electrical stimulation, and neuromuscular facilitation techniques, whereas CM-based rehabilitation encompasses acupuncture and other traditional modalities (3). Nevertheless, prolonged treatment courses and suboptimal patient adherence frequently limit their clinical effectiveness. Accordingly, the identification of adjunct therapies that are effective, convenient, and safe holds substantial clinical significance.

With the advancement of CM, there has been increasing research into the efficacy of various acupuncture techniques for post-stroke ULMD. Commonly employed modalities include body acupuncture (BA), scalp acupuncture (SA), electroacupuncture (EA), warm acupuncture (WA), and wrist ankle needle (WAN). Increasing literature has examined these interventions, with some studies focusing specifically on the therapeutic effects of individual acupuncture methods. For instance, a meta-analysis by Li Hongpei demonstrated that combining scalp and BA with conventional rehabilitation effectively ameliorates upper limb spastic hemiplegia following cerebral infarction (4). Similarly, Zhang Dongxue’s meta-analysis highlighted the superiority of SA combined with rehabilitation in reducing spasticity and ameliorating limb function (5). Wu Linlu’s network meta-analysis (NMA) further revealed that rehabilitation combined with SA was the most effective intervention for improving total Fugl-Meyer Assessment (FMA) scores, while rehabilitation combined with EA achieved the highest surface under the cumulative ranking curve (SUCRA) values for improving the upper limb subscale of the FMA (6). Nevertheless, few studies have evaluated newer techniques, such as WAN or fire needle (FN), and there remains no consensus regarding the optimal acupuncture modality for post-stroke ULMD.

In light of the absence of standardized, evidence-based interventions, an NMA was performed to systematically assess the comparative efficacy of different acupuncture methods for post-stroke ULMD by integrating both direct and indirect evidence. This study aimed to compare the relative efficacy of different acupuncture modalities and acupuncture-related combination therapies for post-stroke upper limb motor dysfunction, and to provide comparative evidence regarding their potential adjunctive role in rehabilitation practice, while acknowledging limitations in generalizability.

## 2 Method

### 2.1 Design and registration

This NMA followed the Preferred Reporting Items for Systematic Reviews and Meta-Analyses (PRISMA) statement. The study was registered in the International Prospective Register of Systematic Reviews (PROSPERO; identifier: CRD42024589860).

### 2.2 Search methods

The researchers independently searched PubMed, EMBASE, Cochrane Library, Web of Science, CNKI, SinoMed, China Science and Technology Journal Database, and Wanfang. The search period spanned from database inception to August 29, 2024. No restrictions on region or language were applied during the literature search. Keywords encompassed “acupuncture,” “EA,” “stroke,” “upper limb,” and “paralysis,” along with all corresponding synonyms. To avoid omission of relevant studies, references of pertinent literature were also manually reviewed. The strategies are detailed in Supplementary File 1.

### 2.3 Eligibility criteria

The study strictly adhered to the PICOS eligibility principles. Inclusion criteria were: (1) Adult patients diagnosed with stroke, including hemorrhagic and ischemic subtypes, accompanied by upper limb or motor dysfunction; (2) Intervention groups receiving at least one form of acupuncture therapy: BA, EA, FN, SA, WAN, or WA; (3) Control groups receiving either placebo or standard treatment. If both intervention and control groups received concurrent general adjuvant therapy, the therapy had to be identical; (4) Studies reporting at least one of the following outcomes: motor function (MF) [FMA, Fugl-Meyer Assessment of Upper Extremity (FMA-UE)], activities of daily living (ADL) [Modified Barthel Index (MBI), Barthel Index (BI)], muscle spasticity [Modified Ashworth Scale (MAS), Clinical Spasticity Index (CSI)], or pain intensity [Visual Analogue Scale (VAS)]; (5) randomized controlled trials (RCTs) with available full texts. No restrictions on region or language were applied during the search process.

Exclusion criteria were: (1) Post-stroke patients without ULMD; (2) Studies not involving acupuncture; (3) Case reports, animal experiments, studies involving minors, or conference abstracts; (4) Single-arm trials.

### 2.4 Study selection

As per the foregoing predefined eligibility criteria, two researchers (H.Y.Y. and S.L.T.) independently selected eligible studies. All potentially relevant records were imported into EndNote 21 to remove duplicates. Titles and abstracts were checked to remove ineligible studies, followed by full-text review. Dissents were addressed via discussion or consultation with a third reviewer (Y.J.L.).

### 2.5 Data extraction and quality assessment

Extracted data encompassed: (1) Basic study information: title, first author, and year of publication; (2) Characteristics of study participants: mean age, sex distribution, number of participants per group, disease duration, and baseline functional status where available (e.g., baseline FMA/FMA-UE, ADL, or spasticity scores). (3) Details of interventions: specific measures and timing; (4) Outcome measures: FMA, MBI, and MAS scores. Data were extracted by one reviewer and verified for accuracy by a second reviewer. Risk of bias was independently assessed by two reviewers via the Cochrane Risk of Bias Tool version 2 (RoB 2.0) for randomized trials (7). Domains evaluated encompassed bias arising from the randomization process, deviations from intended interventions, missing outcome data, outcome measurement, and selection of reported results, including deviations from the registered protocol. Given the inherent challenges of blinding in acupuncture trials, the lack of blinding of participants and personnel was primarily considered under the domain of deviations from intended interventions, whereas potential detection bias was assessed under the domain of outcome measurement. Studies with one or more domains rated as “high risk” were classified as overall high risk of bias; studies with all domains rated as “low risk” were classified as overall low risk of bias. Cross-checking was performed, and disagreements were resolved via consultation with a third reviewer.

### 2.6 Data synthesis and statistical analysis

Bayesian statistical models were constructed using JAGS software (gemtc 0.8–2 and rjags 4–10 packages) in R (version 4.4.2, RStudio, Boston, MA, United States). Continuous outcomes were shown in standardized mean differences (SMDs) with 95% credible intervals (CrIs), and categorical outcomes were shown in pooled risk ratios (RRs) with 95% CrIs. Random-effects models were applied due to clinical heterogeneity among trials (differences in country, acupuncture modality, and disease severity). The SUCRA was utilized to estimate the relative ranking of various acupuncture interventions for each outcome; higher SUCRA values indicate a higher rank. Consistency and inconsistency models were compared using the deviance information criterion (DIC); a DIC difference of less than 5 indicated good consistency, and the consistency model was employed. Global inconsistency was evaluated via the node-splitting approach, which compares direct and indirect evidence; a *p*-value >0.05 indicates statistically insignificant inconsistency. Network plots and comparison-adjusted funnel plots were generated via Stata 15.0 (StataCorp, College Station, TX, United States).

### 2.7 Clinical recommendations and grading criteria

The certainty of evidence was evaluated using the Confidence in Network Meta-Analysis (CINeMA) framework, based on the GRADE approach, across six domains: within-study bias, reporting bias, indirectness, imprecision, heterogeneity, and incoherence. The overall confidence for each comparison was rated as high, moderate, low, or very low, and was used to inform clinical recommendations according to outcome and treatment duration.

## 3 Results

### 3.1 Search outcomes

**Figure 1:**
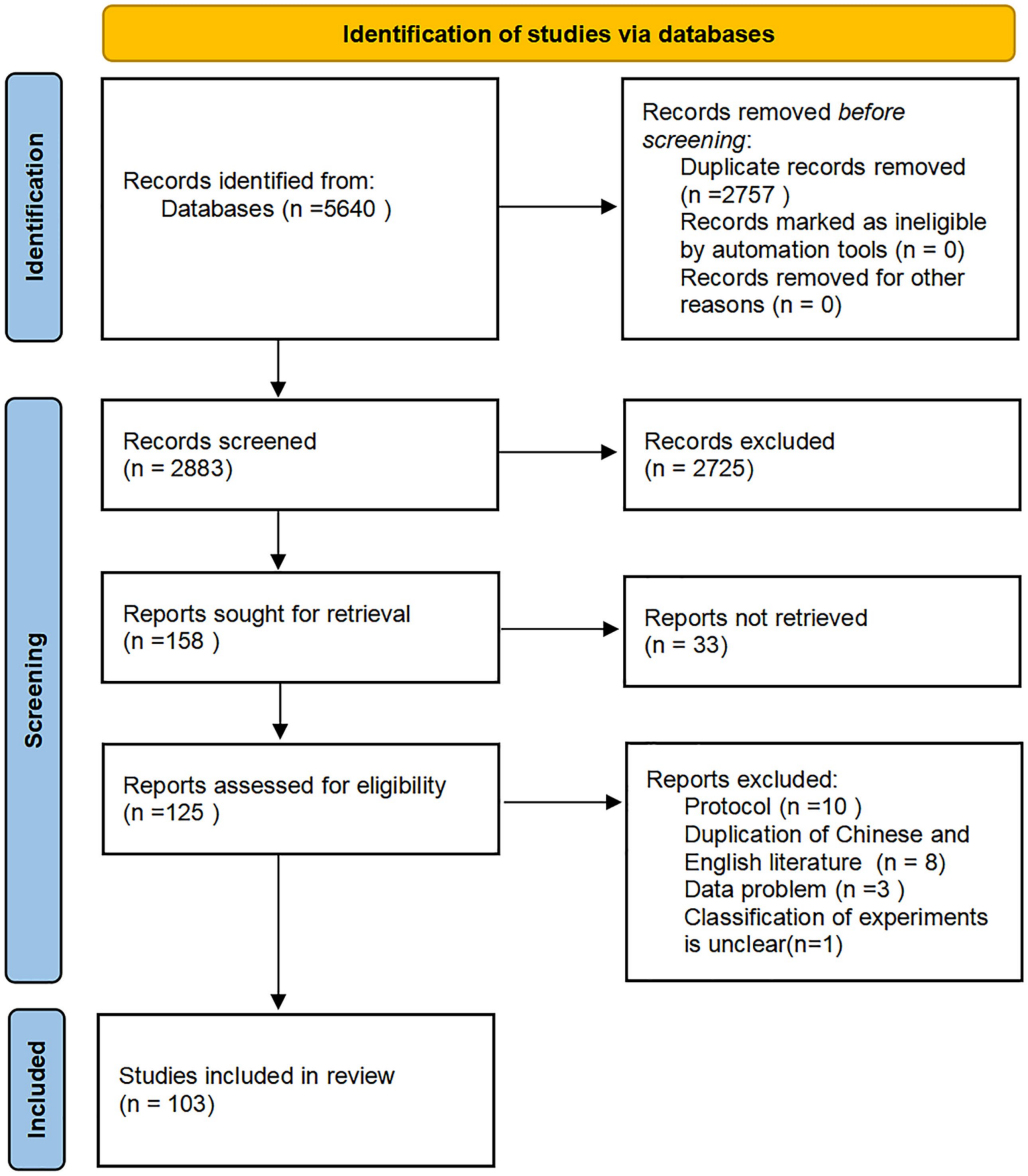
PRISMA flow diagram for search and selection of eligible studies encompassed in the NMA.

The detailed procedure for study selection is illustrated in the PRISMA flow diagram (Figure 1). Initially, 5,640 potentially pertinent studies were identified across eight databases. Subsequently, 2,757 duplicate studies were removed. Titles and abstracts were subsequently screened as per eligibility criteria, resulting in the exclusion of an additional 2,725 studies. Among the remaining 158 articles, 33 were unavailable for full-text retrieval. Of the retrieved literature, 22 studies were excluded due to data issues, document type, duplication of Chinese and English articles, or unclear experimental classification. Ultimately, 103 articles were deemed eligible.

### 3.2 Characteristics of encompassed studies

The characteristics of the encompassed studies are summarized in abbreviations section. Among the 103 eligible studies published between 2015 and 2024, one study was conducted in Spain [36] and 102 in China [1–35, 37–103]. Ten studies utilized multicenter databases [2, 25, 35–36, 61–63, 91, 94, 96]. Overall, 9,351 patients participated across the 103 RCTs, with ages of 48.4–73.7. The analysis encompassed four studies on FN, two studies on WAN, 19 studies on EA, one study on warm needle therapy, and 22 studies on SA. Baseline functional status was inconsistently reported across studies, indicating additional clinical heterogeneity among the included populations.

### 3.3 Quality assessment

**Figure 2:**
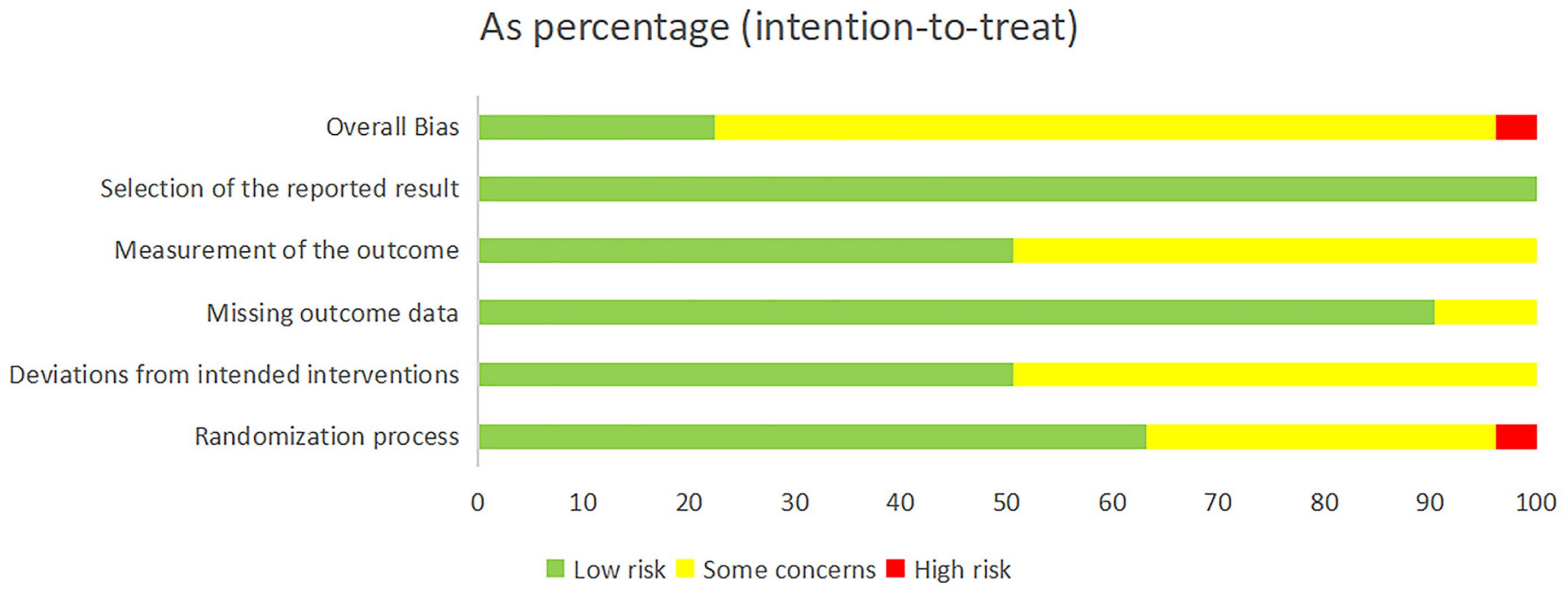
Risk of bias graph.

The risk of bias assessment is presented in Figure 2. Most RCTs (*n* = 103, 100%) demonstrated a low risk of bias regarding the selection of reported results. Nevertheless, a minority of trials exhibited bias in outcome measurement (*n* = 5, 4.9%), bias due to missing outcome data (*n* = 10, 9.7%), deviations from intended interventions (*n* = 51, 49.5%), randomization process (*n* = 34,33%), and the measurement of the outcome (*n* = 51,49.5%). A small proportion of trials (*n* = 4, 3.9%) showed a high risk of bias in randomization owing to non-random allocation of participants. Detailed evaluation results are provided in Supplementary Figure S1.

### 3.4 NMA

#### 3.4.1 ADL (>1 month)

**Figure 3:**
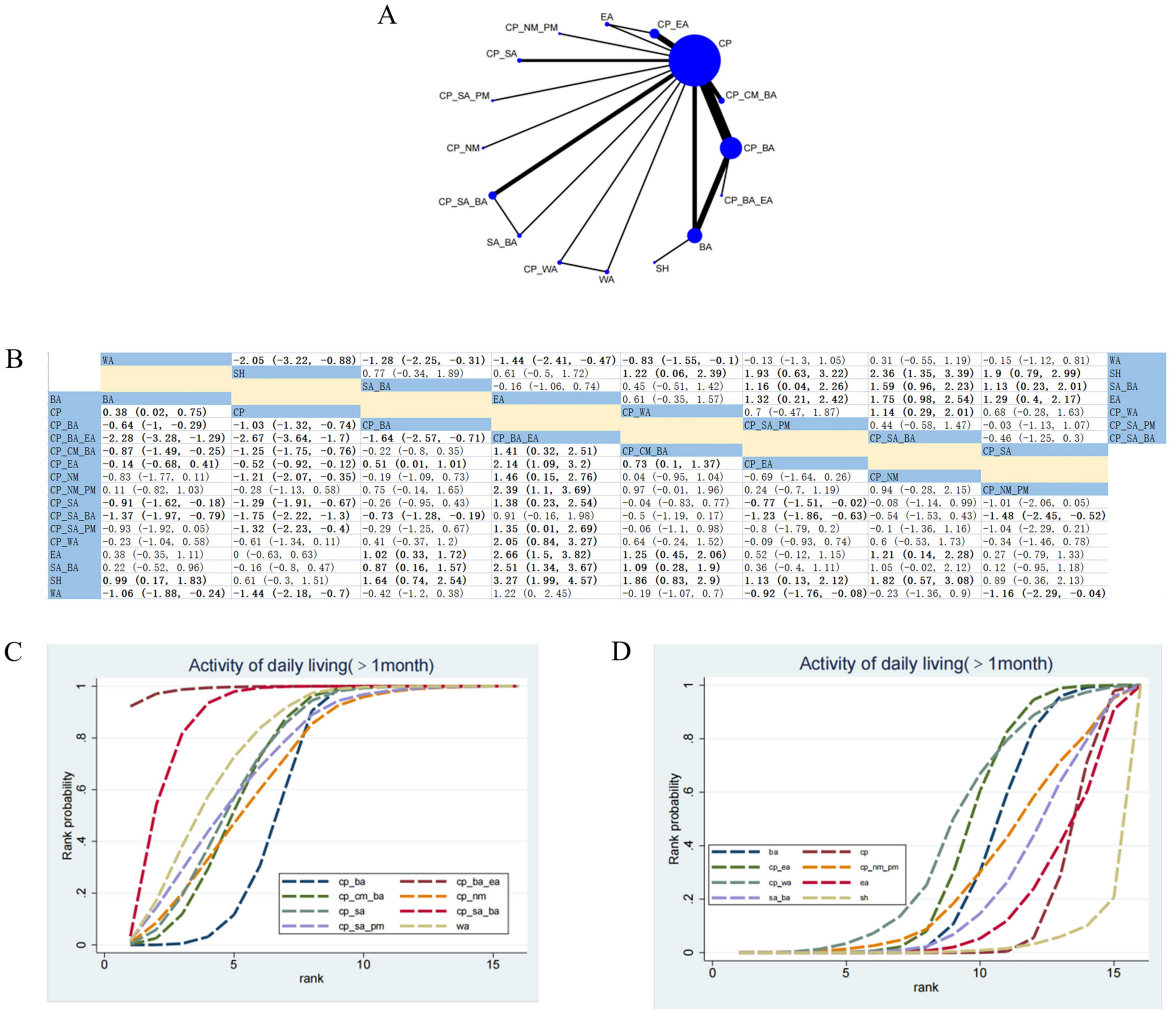
Network plot and results of NMA for the ADL (>1 month). **(A)** Network plot. **(B)** Relative effectiveness of therapies; estimates are shown in SMD with 95% CrI. Comparisons should be interpreted from left to right; significant results are in bold. **(C,D)** Cumulative probabilities for different interventions; **(C)** illustrates the eight highest-ranked treatments based on SUCRA, and **(D)** the eight lowest-ranked interventions.

26 RCTs assessed the effects of 16 therapeutic interventions (Figure 3A). Compared with SH, CP_CM_BA (SMD 1.86; 95% CrI [0.83, 2.9]), CP_NM (SMD 1.82; 95% CrI [0.57, 3.08]), and CP_BA_EA (SMD 3.27; 95% CrI [1.99, 4.57]) demonstrated significant advantages in improving ADL (Figure 3B). Based on SUCRA values, CP_BA_EA (SUCRA = 99.13%) ranked as the most effective treatment (Figures 3C,D; Supplementary Table S1). According to CINeMA assessments, the confidence in evidence was low for comparisons of CP_BA_EA versus SH, CP_CM_BA versus SH, and CP_NM versus SH (Supplementary Table S3).

#### 3.4.2 ADL (≤1 month)

**Figure 4:**
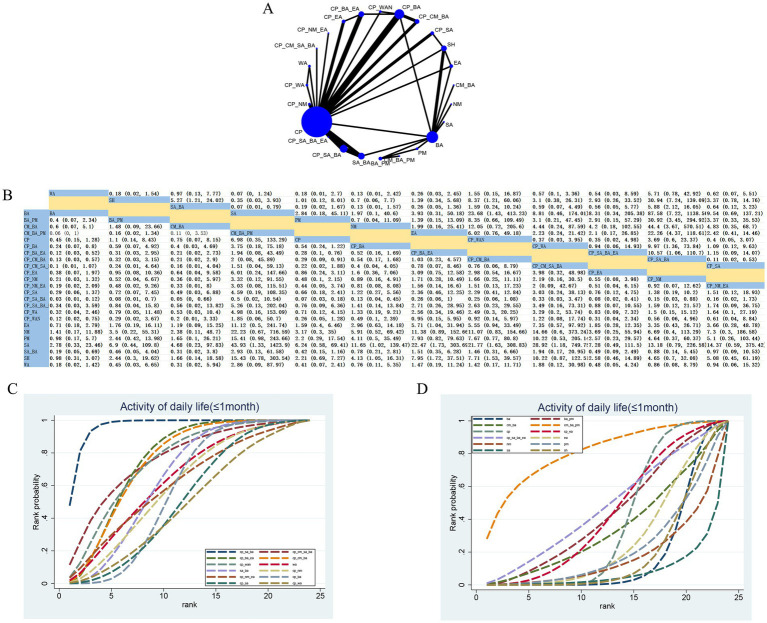
Network plot and results of NMA for ADL (≤1 month). **(A)** Network plot. **(B)** Relative effectiveness of therapies; estimates are shown in SMD with 95% CrI. Comparisons should be interpreted from left to right; significant results are in bold. **(C)** Cumulative probabilities for different interventions; (C) illustrates the twelve highest-ranked treatments based on SUCRA, and **(D)** the twelve lowest-ranked interventions.

39 RCTs evaluated 24 therapeutic interventions (Figure 4A). Compared with BA and CP, CP_SA_BA was associated with more favorable network estimates of 0.03 (95% CrI: 0.01 to 0.12) and 0.07 (95% CrI: 0.03 to 0.18), respectively (Figure 4B). According to SUCRA, CP_SA_BA(SUCRA=96.43%) was identified as the optimal treatment (Figures 4C,D; Supplementary Table S1). Moderate confidence was observed for CP versus CP_SA_BA and BA versus CP (Supplementary Table S3).

#### 3.4.3 MF (>1 month)

**Figure 5:**
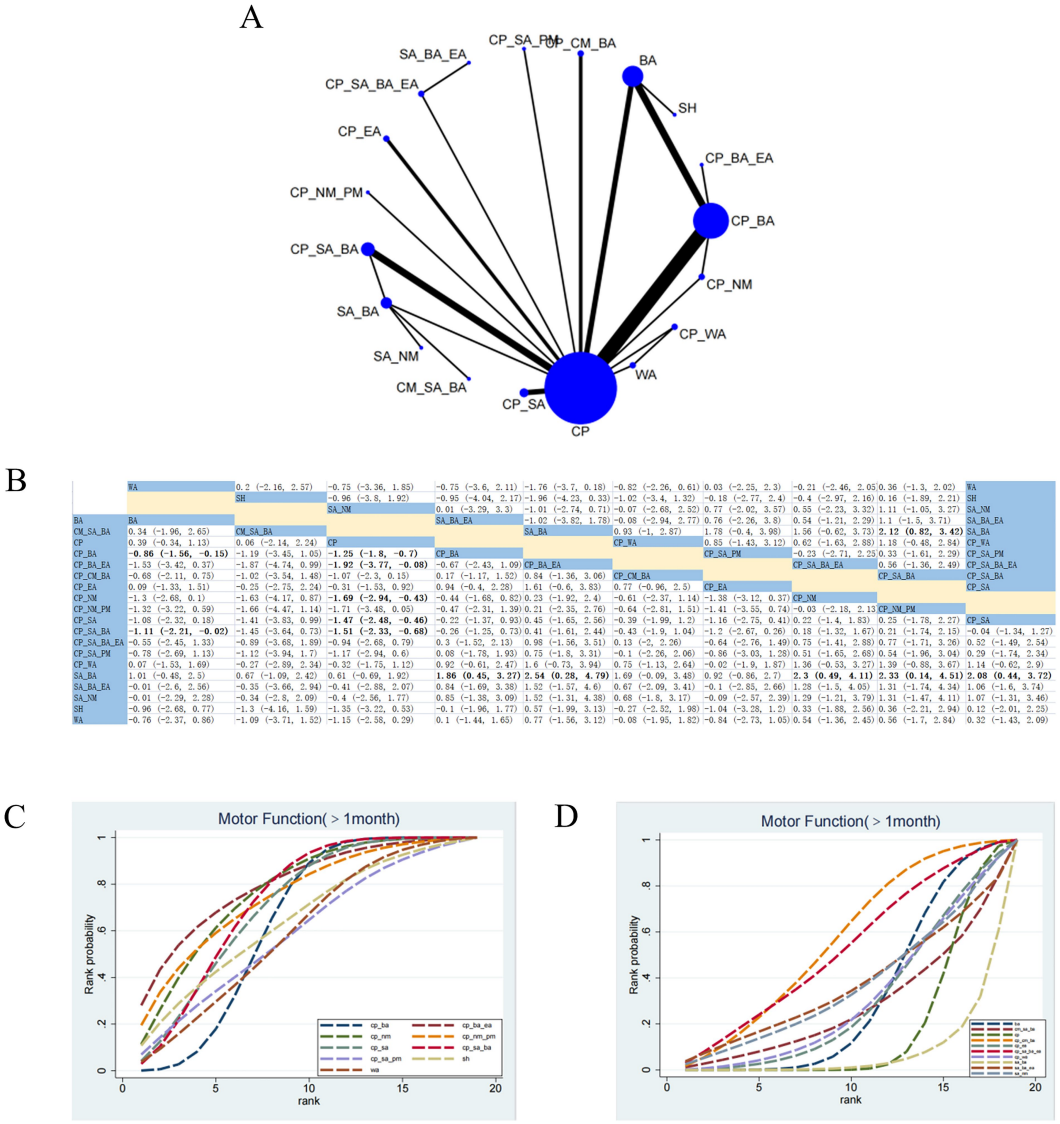
Network plot and results of NMA for MF (>1 month). **(A)** Network plot. **(B)** Relative effectiveness of therapies; estimates are shown in SMD with 95% CrI. Comparisons should be interpreted from left to right; significant results are in bold. **(C,D)** Cumulative probabilities for different interventions; **(C)** illustrates the highest-ranked treatments, and **(D)** the lowest-ranked interventions.

31 RCTs evaluated 17 therapeutic interventions (Figure 5A). Compared with SA_BA, CP_BA_EA (SMD 2.54; 95% CrI [0.28, 4.79]), CP_NM (SMD 2.3; 95% CrI [0.49, 4.11]), CP_SA_BA (SMD 2.12; 95% CrI [0.82, 3.42]), CP_SA (SMD 2.08; 95% CrI [0.44, 3.72]), and CP_NM_PM (SMD 2.33; 95% CrI [0.14, 4.51]) demonstrated significant improvements in MF (Figure 5B). Based on SUCRA, CP_BA_EA (SUCRA = 79.67%) ranked as the most effective intervention (Figures 5C,D; Supplementary Table S1). Low confidence was identified for CP_BA_EA versus SA_BA, while very low confidence was observed for CP_BA_EA versus CP_BA (Supplementary Table S3).

#### 3.4.4 MF (≤1 month)

**Figure 6:**
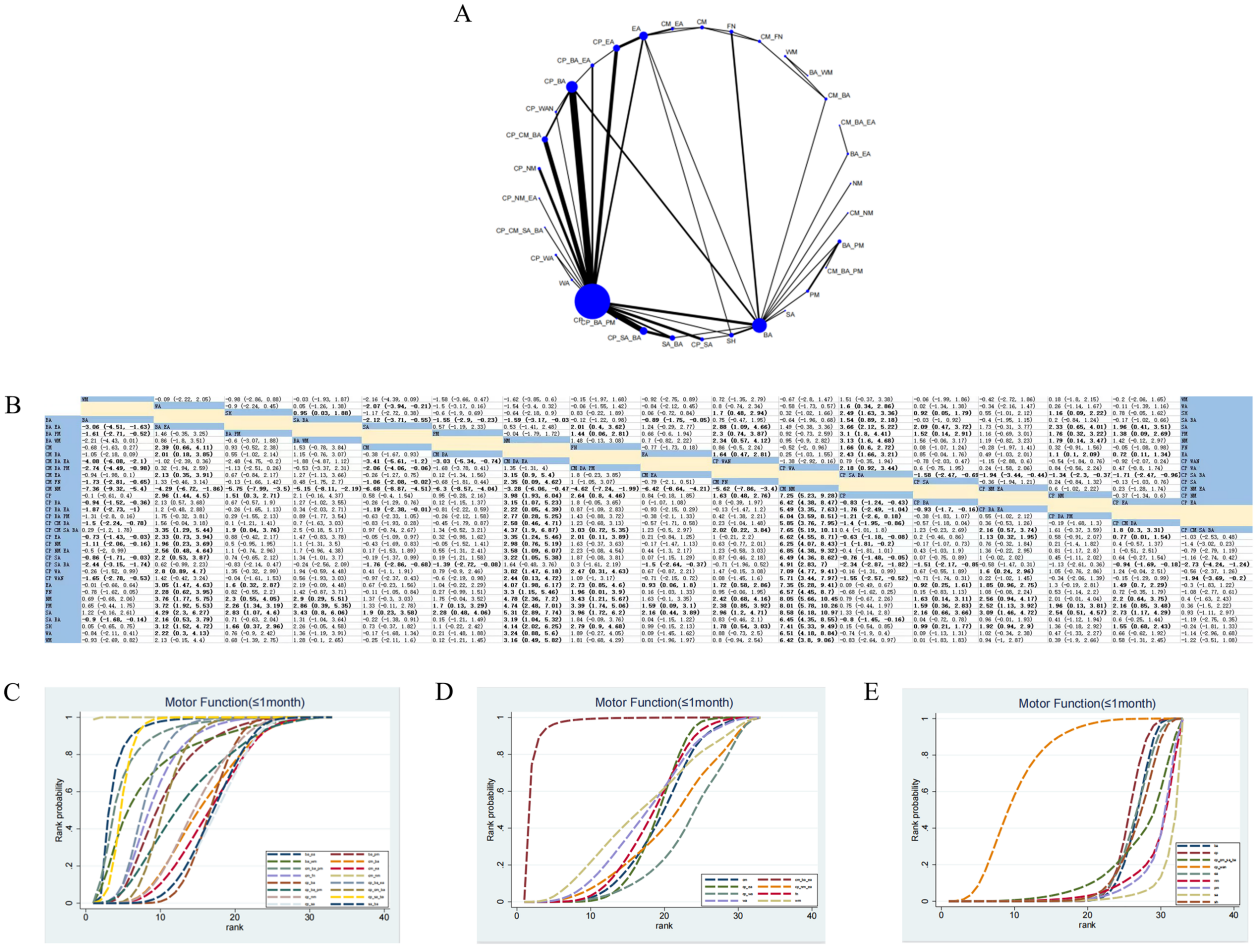
Network plot and NMA results. **(A)** Network plot for MF (≤1 month). **(B)** Relative effectiveness of therapies on MF (≤1 month). Estimates are shown in SMD with 95% CrI in parentheses. Comparisons should be interpreted from left to right; treatment effects are evaluated at the intersection of the corresponding row and column, with significant results highlighted in bold. **(C–E)** Cumulative probability of different treatment modalities for MF (≤1 month). **(C)** Depicts the nine highest-ranked interventions based on SUCRA values; **(D)** shows the eight mid-ranked interventions; **(E)** illustrates the nine lowest-ranked interventions.

61 RCTs evaluated the effects of 33 therapeutic approaches (Figure 6A). Compared with CM_NM, CP_BA_EA (SMD: −3.28; 95% CrI −6.06, −0.47) and BA_EA (SMD: −4.29; 95% CrI −6.72, −1.86) were the closest in effect. In contrast, compared with SA (SMD: 8.58; 95% CrI [6.18, 10.97]) and NM (SMD: 8.05; 95% CrI [5.66, 10.45]), CM_NM exhibited significant superiority in improving MF (Figure 6B). According to SUCRA, CM_NM (SUCRA = 99.96%) demonstrated the most favorable effect on MF compared with other treatment modalities (Figures 6C–E; Supplementary Table S1). CINeMA indicated moderate confidence for CP versus CP_SA_BA, low confidence for BA versus CP_SA_BA, and low confidence for CP_SA_BA versus SA_BA (Supplementary Table S3).

#### 3.4.5 Muscle cramp severity (>1 month)

**Figure 7:**
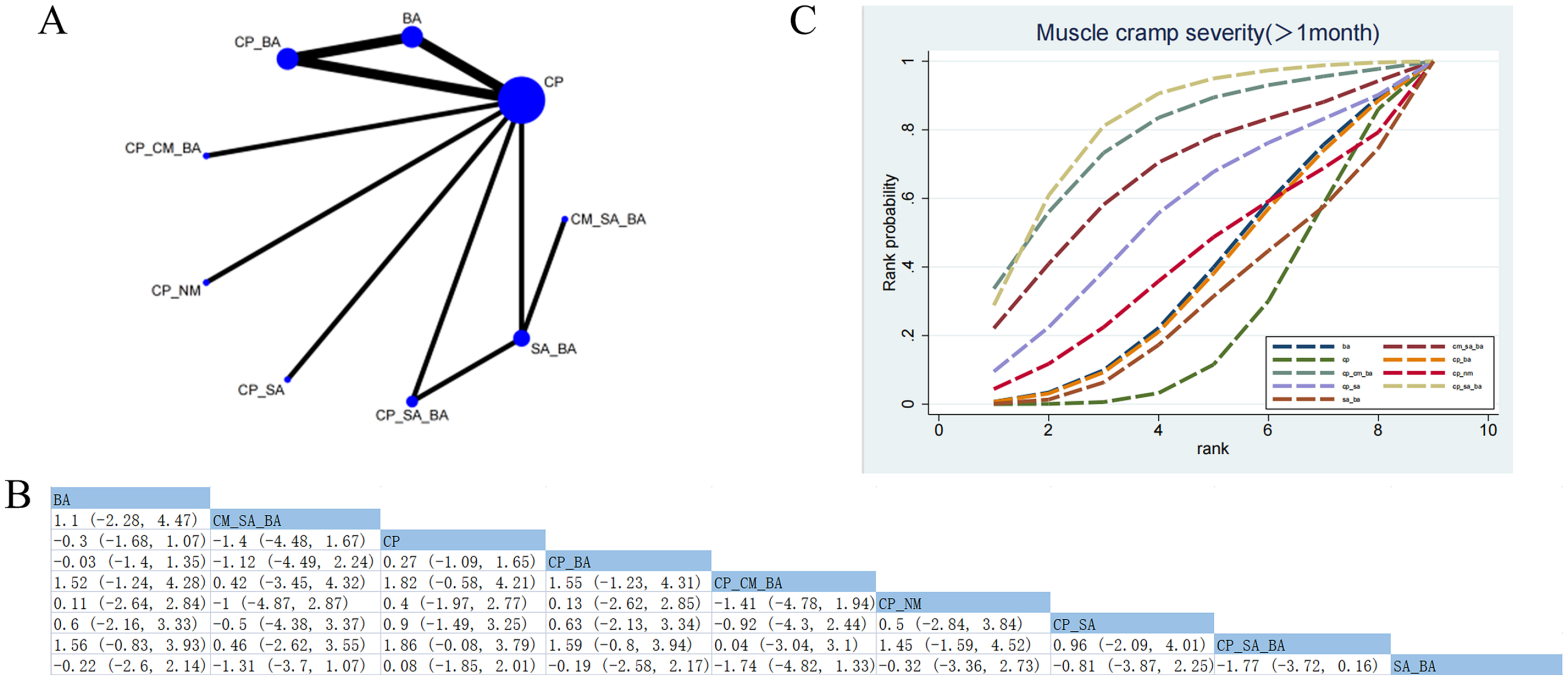
Network plot and NMA results. **(A)** Network plot for muscle cramp severity (>1 month). **(B)** Relative effectiveness of therapies on muscle cramp severity (>1 month). Estimates are shown in SMD and 95% CrI in parentheses. Comparisons between treatment options should be viewed from left to right. Treatment effects are evaluated at the intersection of the option’s column and row; significant results appear in bold. **(C)** Cumulative probability of different treatment modalities for muscle cramp severity (>1 month).

Seven RCTs assessed the effects of nine treatment approaches (Figure 7A). No statistically significant differences were observed in pairwise comparisons among the interventions (Figure 7B). Based on SUCRA, CP_SA_BA (SUCRA = 81.53%) demonstrated the most favorable effect in relieving muscle tension (Figure 7C; Supplementary Table S1). Based on CINeMA, all corresponding comparisons, including CP versus CP_SA_BA, CP_SA_BA versus SA_BA, and BA versus CP_SA_BA, were rated as low confidence (Supplementary Table S3).

#### 3.4.6 Muscle cramp severity (≤1 month)

**Figure 8:**
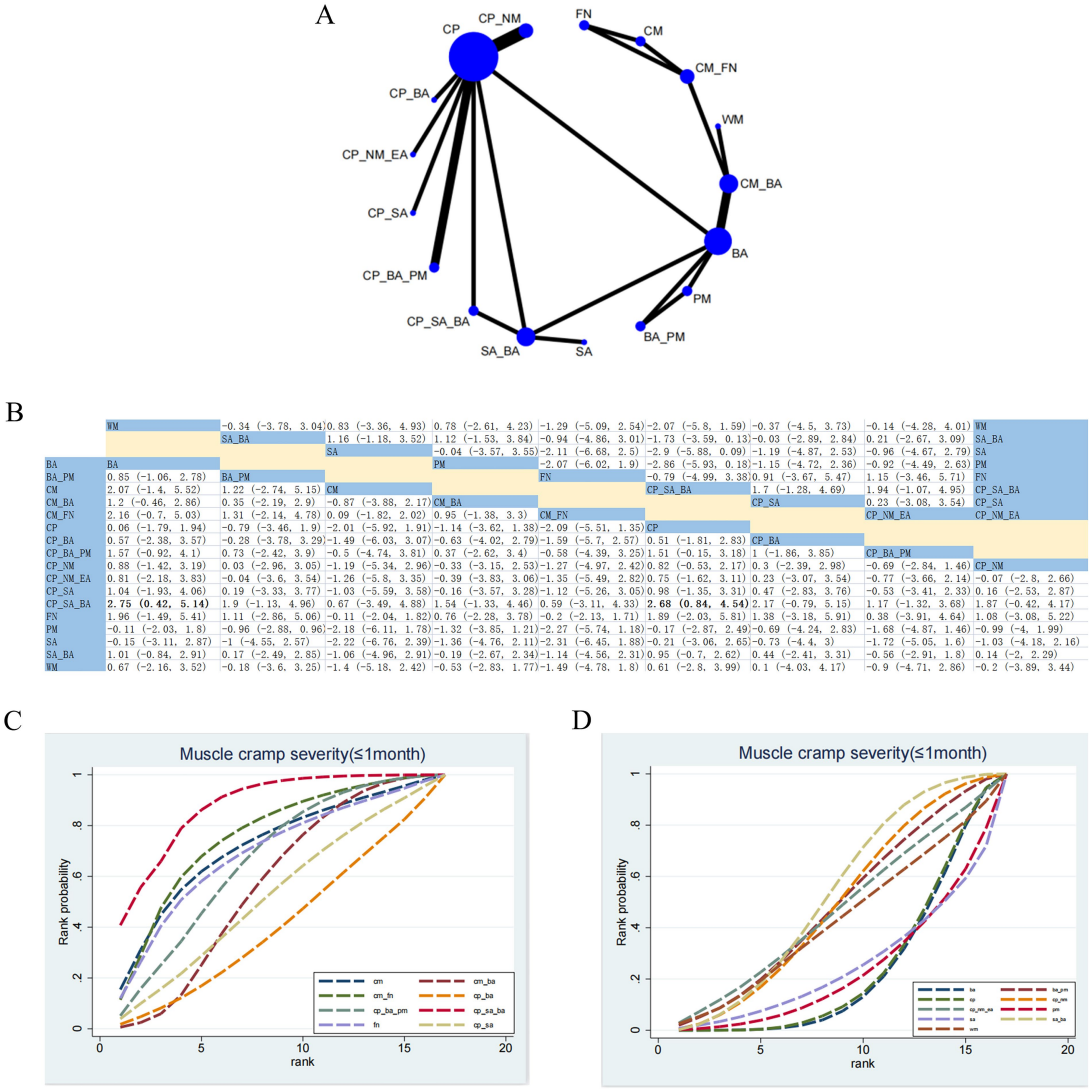
Network plot and NMA results. **(A)** Network plot for muscle cramp severity (≤1 month). **(B)** Relative effectiveness of therapies. Estimates are shown in SMD and 95% CrI in parentheses. Comparisons between treatment options should be viewed from left to right. Treatment effects are evaluated at the intersection of the option’s column and row; significant results appear in bold. **(C,D)** Cumulative probability of different treatment modalities. **(C)** Illustrates the eight highest-ranked interventions based on SUCRA; **(D)** shows the nine lowest-ranked interventions.

18 RCTs assessed the effects of 17 therapeutic approaches (Figure 8A). Compared with CP_SA_BA, CP (SMD: 2.68; 95% CrI [0.84, 4.54]) and BA (SMD: 2.75; 95% CrI [0.42, 5.14]) showed less favorable effects in alleviating muscle spasm (Figure 8B). Based on SUCRA, CP_SA_BA (SUCRA = 87.77%) exhibited the most significant advantage in relieving muscle tension (Figures 8C, D; Supplementary Table S1). CINeMA indicated low confidence for CP versus CP_SA_BA, low confidence for BA versus CP_SA_BA, and low confidence for CP_SA_BA versus SA_BA (Supplementary Table S3).

#### 3.4.7 VAS

**Figure 9:**
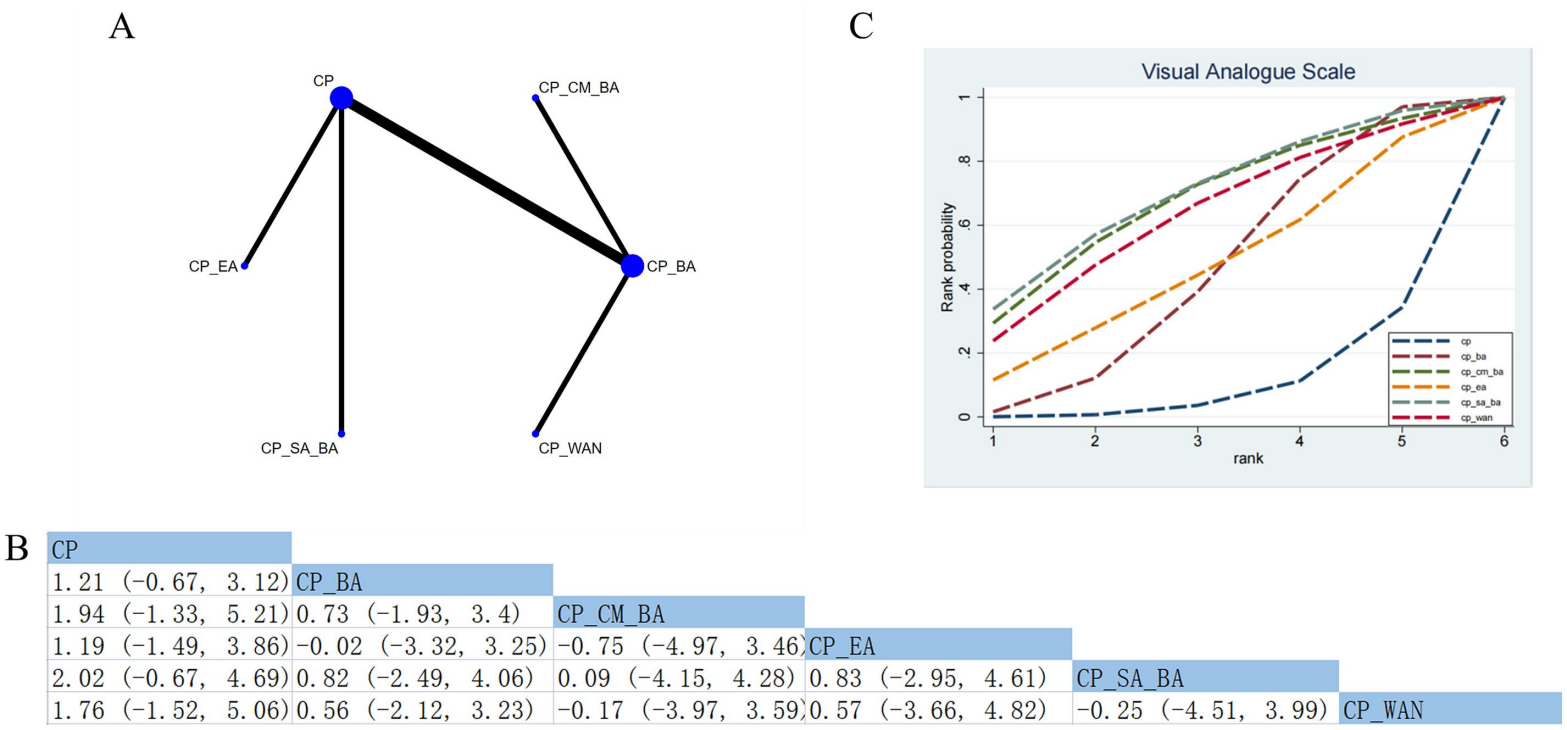
Network plot and NMA results. **(A)** Network plot for VAS. **(B)** Relative effectiveness of therapies. Estimates are shown in mean difference (MD) and 95% CrI in parentheses. Comparisons between treatment options should be viewed from left to right. Treatment effects are evaluated at the intersection of the option’s column and row; significant results appear in bold. **(C)** The cumulative probability concerning different treatment modalities for VAS.

Six RCTs assessed the effects of 6 therapeutic approaches (Figure 9A). No statistically significant differences were found in pairwise comparisons among the interventions (Figure 9B). Based on SUCRA, CP_SA_BA (SUCRA = 69.24%) demonstrated a significant advantage in pain relief (Figure 9C; Supplementary Table S1). For VAS, a formal certainty assessment using CINeMA was not feasible due to the sparsity of the evidence network, with only a limited number of studies and several comparisons informed by single trials. Therefore, although network estimates for this outcome were reported, the certainty of evidence could not be formally evaluated, and these findings should be interpreted with caution.

#### 3.4.8 Clinical response rate (CRR)

**Figure 10:**
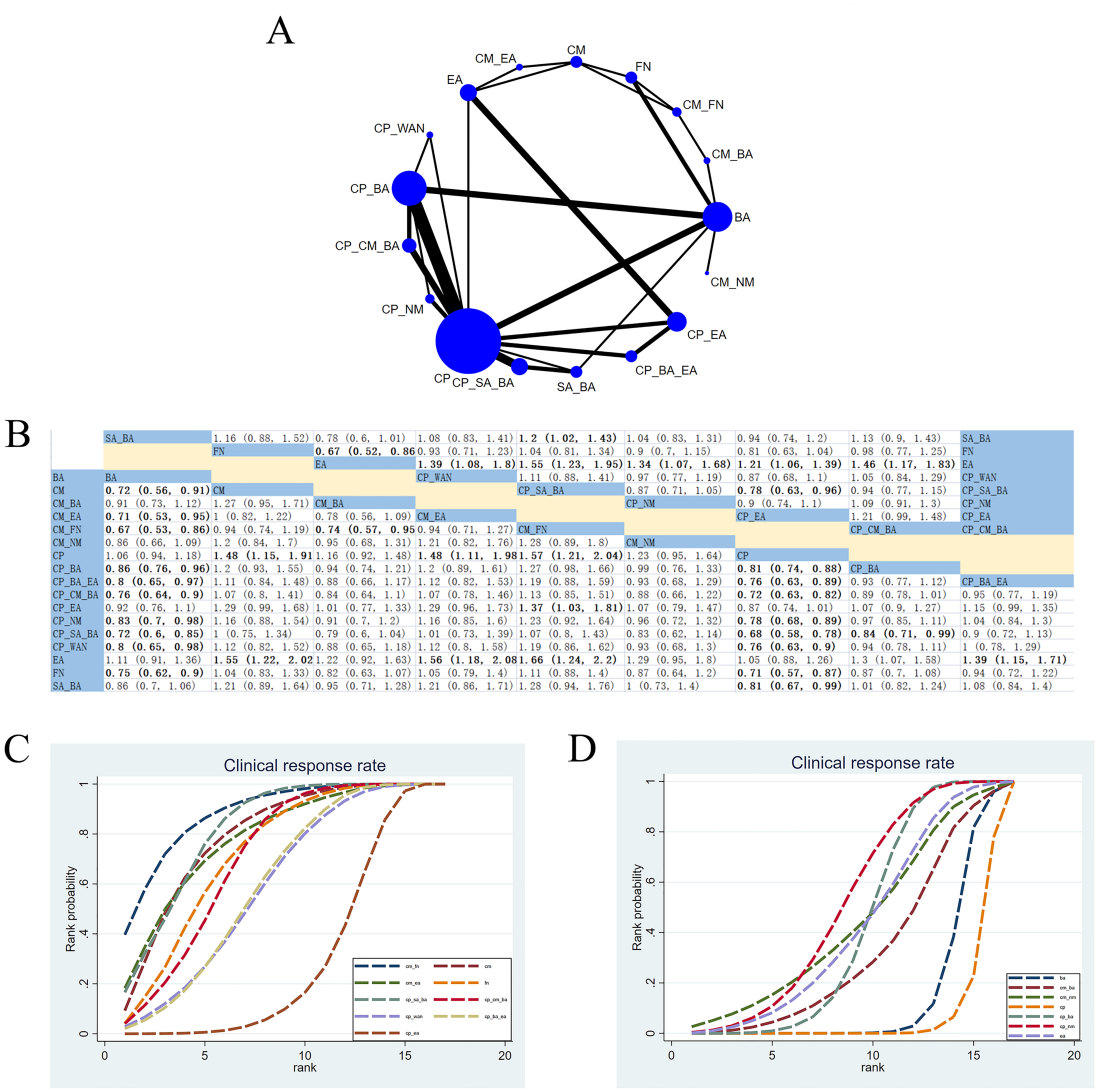
Network plot and NMA results. **(A)** Network plot for CRR. **(B)** Relative effectiveness of therapies. As above; estimates shown in RR with 95% CrI. Estimates are shown in RR and 95% CrI in parentheses. Comparisons between treatment options should be viewed from left to right. Treatment effects are evaluated at the intersection of the option’s column and row; significant results appear in bold. **(C,D)** The cumulative probability concerning different treatment modalities for CRR. Among them, **(C)** illustrates the nine highest-ranked interventions based on SUCRA, whereas **(D)** shows the nine lowest-ranked interventions in the cumulative ranking analysis.

37 RCTs assessed the effects of 17 therapeutic approaches (Figure 10A). Compared with EA, CM_FN (RR: 1.66; 95% CrI [1.24, 2.2]), CM_EA (RR: 1.56; 95% CrI [1.18, 2.08]), CP_SA_BA (RR: 1.55; 95% CrI [1.23, 1.95]) and CM (RR: 1.55; 95% CrI [1.22, 2.02]) were the most effective in improving CRR. Compared with CP, CM_FN (RR: 1.57; 95% CrI [1.21, 2.04]), CM_EA (RR: 1.48; 95% CrI [1.11, 1.98]), and CM (RR: 1.48; 95% CrI [1.15, 1.91]) were the top three (Figure 10B). According to SUCRA, CM_FN (SUCRA = 88.11%) demonstrated the most favorable improvement in clinical efficiency (Figures 10C, D; Supplementary Table S1). CINeMA indicated high confidence for CM_FN versus EA and CM_FN versus CP; moderate confidence for CM_EA versus EA, CP_SA_BA versus EA, CM_EA versus CP, and CM versus EA (Supplementary Table S3).

#### 3.4.9 Progression rate to grade 0 on the MAS

**Figure 11:**
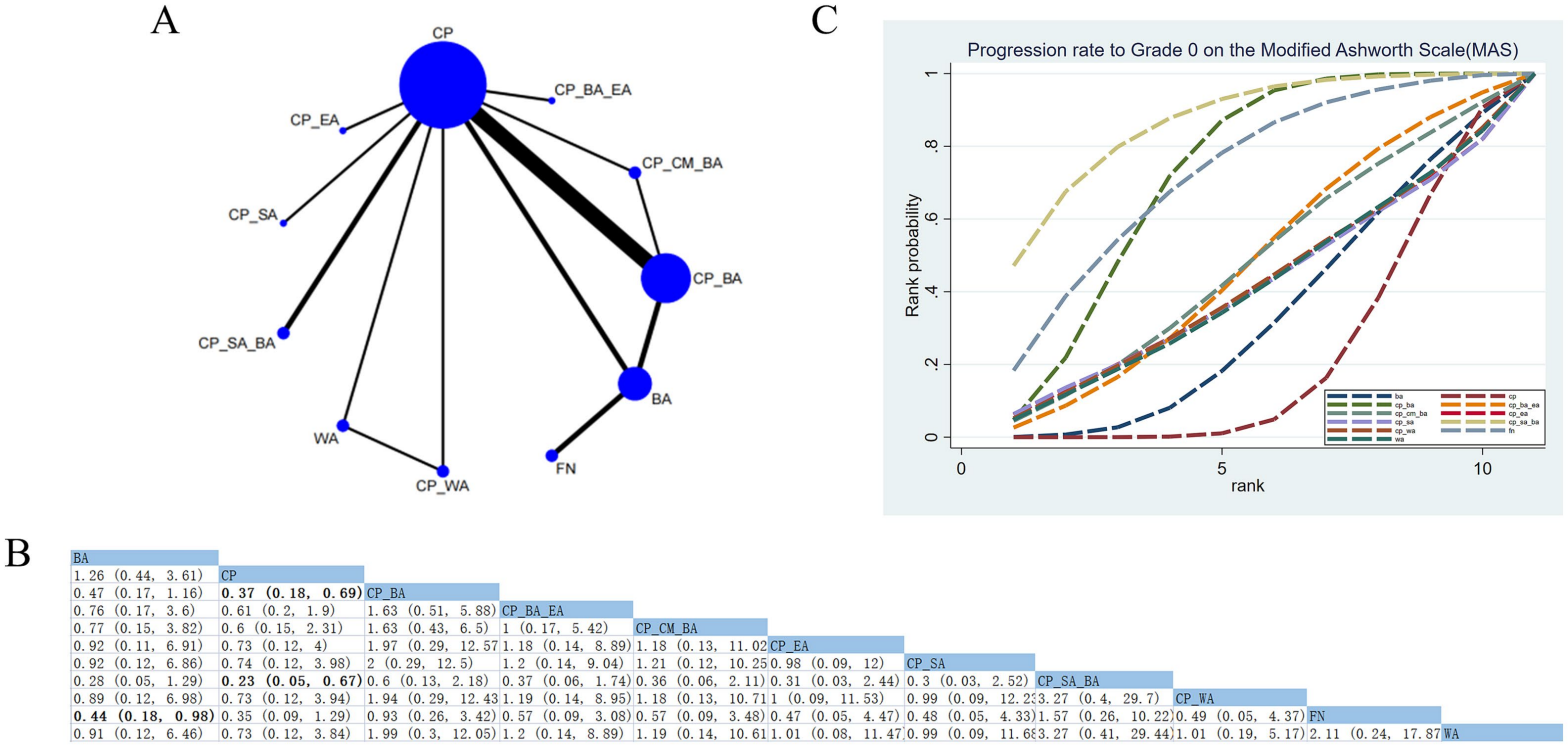
Network plot and NMA results. **(A)** Network plot for progression rate to Grade 0 on the MAS. **(B)** Relative effectiveness of therapies on progression rate to Grade 0 on the MAS. Estimates are shown in RR and 95% CrI in parentheses. Comparisons between treatment options should be viewed from left to right. Treatment effects are evaluated at the intersection of the option’s column and row; significant results appear in bold. **(C)** The cumulative probability concerning different treatment modalities for the progression rate to Grade 0 on the MAS.

18 RCTs assessed the effects of 11 therapeutic approaches (Figure 11A). Compared with FN, BA (RR: 0.44; 95% CrI [0.18, 0.98]) was less effective in improving muscle strength. Similarly, conventional physical therapy (CP) (RR: 0.23; 95% CrI [0.05, 0.67]) was less effective than CP_SA_BA (Figure 11B). Based on SUCRA, CP_SA_BA (SUCRA = 86.9%) demonstrated a significant advantage (Figure 11C; Supplementary Table S1). Based on CINeMA, moderate confidence was observed for CP_SA_BA versus CP and BA versus FN, whereas low confidence was found for CP_SA_BA versus FN (Supplementary Table S3).

#### 3.4.10 Spasm relief effectiveness rate

**Figure 12:**
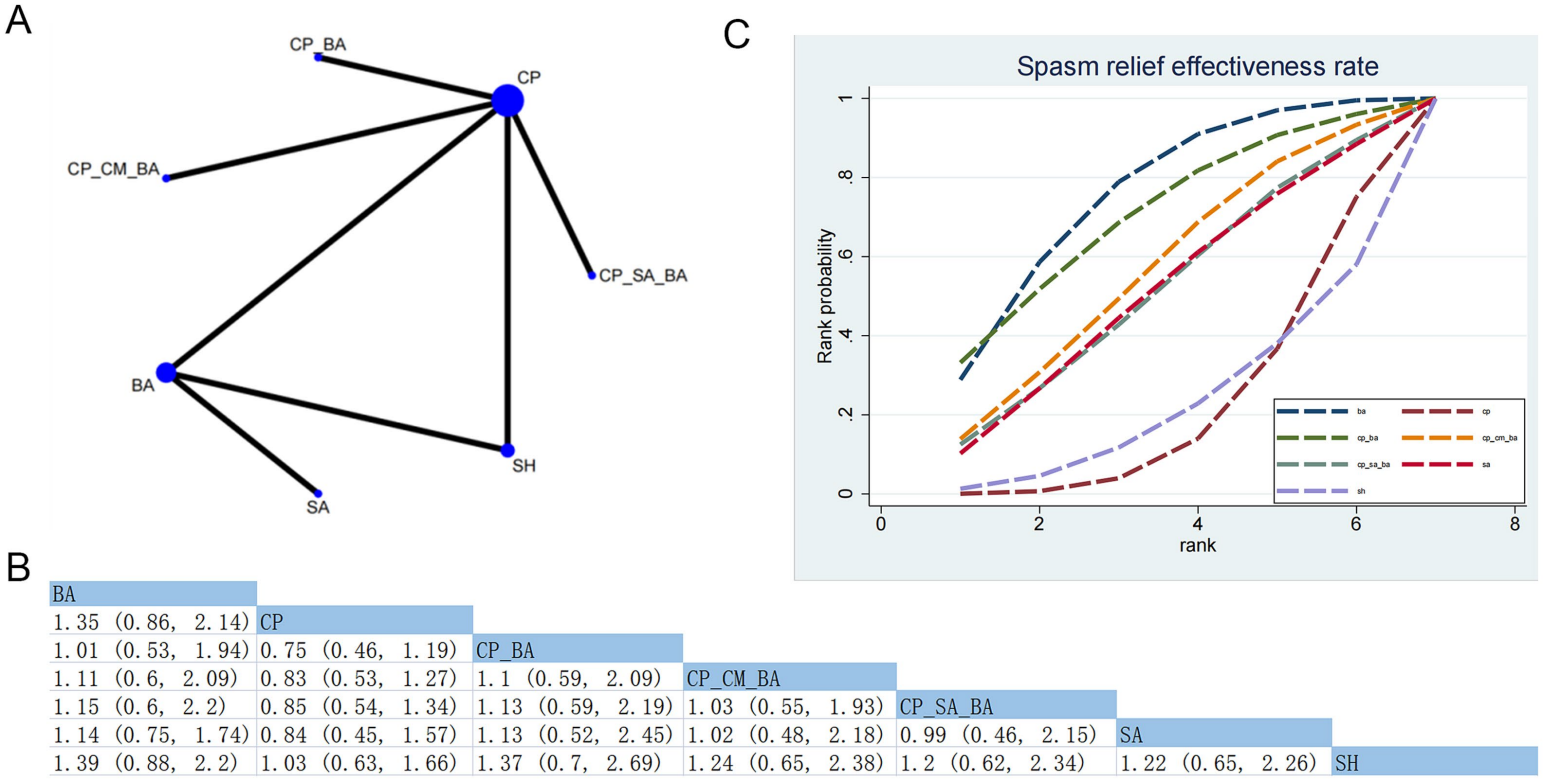
Network plot and results of the NMA. **(A)** Network plot for the spasm relief effectiveness rate. **(B)** Relative effectiveness of therapies on the spasm relief effectiveness rate. Estimates are shown in RR with 95% CrI in parentheses. Comparisons between treatment options should be interpreted from left to right. Treatment effects are evaluated at the intersection of the column and row corresponding to the options; statistically significant results are indicated in bold. **(C)** Cumulative probability of different treatment modalities for the spasm relief effectiveness rate.

Five RCTs evaluated the effects of seven therapeutic interventions (Figure 12A). No statistically significant differences were observed in the pairwise comparisons between any of the treatment methods (Figure 12B). Based on SUCRA, BA demonstrated a notable advantage (SUCRA = 75.66%) (Figure 12C; Supplementary Table S1). Moderate confidence was also identified for BA versus SH and BA versus SA (Supplementary Table S3).

### 3.5 Consistency and publication bias assessment

An overall consistency test was conducted for the following outcomes: ADL, MF, muscle cramp severity, VAS, CRR, progression to Grade 0 on the MAS, and spasm relief effectiveness rate. All differences in DIC values were less than 5, indicating good overall consistency. Comparisons derived from the network supported the validity of combining these heterogeneous sources of evidence.

The results (Supplementary Table S1) indicate that various acupuncture interventions establish a well-structured closed-loop intervention model, resulting in significant improvements in ADL (>1 month and ≤1 month), MF (>1 month and ≤1 month), muscle cramp severity (>1 month and ≤1 month), CRR, and progression to Grade 0 on the MAS. The majority of pairwise comparisons showed *p* > 0.05, suggesting good internal consistency and absence of local inconsistency. However, local inconsistencies were identified in the following comparisons: ADL (≤1 month): SA_BA versus BA (*p* < 0.0001), MF (≤1 month): CP versus BA (*p* = 0.02), SA_BA versus BA (*p* = 0.00034), muscle cramp severity (≤1 month): CP versus BA (*p* = 0.01), SA_BA versus BA (*p* = 0.01). Node-splitting results for the remaining outcome measures are provided in Supplementary Table S2. Additionally, adjusted funnel plots were employed to assess publication bias for the outcomes described above, which suggested the presence of publication bias.

## 4 Discussion

A comprehensive search of the published literature was conducted, and evidence from 103 RCTs was analyzed to compare the effects of various acupuncture modalities on MF, ADL, muscle cramp severity, pain intensity, spasm relief effectiveness rate, clinical effectiveness rate, and the improvement rate of grade 0 muscle strength. Regarding MF, CP combined with BA and EA (CP_BA_EA) demonstrated superior efficacy in patients receiving treatment for more than one month, whereas CM combined with NM techniques (CM_NM) was identified as the optimal intervention for those with treatment durations of one month or less. For alleviating muscle cramp severity, CP_SA_BA exhibited the most favorable effect. With respect to ADL, CP_SA_BA was optimal for treatment durations ≤1 month, while CP_BA_EA was preferred when treatment exceeded one month. For pain relief, CP_SA_BA demonstrated the strongest efficacy. CM_FN achieved the highest clinical effectiveness rate. CP_SA_BA was the most effective in improving upper limb muscle strength at grade 0, while BA yielded the highest effectiveness rate for spasm relief.

In research focusing on MF improvement in post-stroke patients, Yang Huinan’s systematic review and meta-analysis of EA for post-stroke shoulder syndrome demonstrated that EA combined with CP and BA achieved superior outcomes compared to EA alone (8). This conclusion corroborates the findings of the present analysis, confirming the therapeutic effect of combined EA and BA. Similarly, in a meta-analysis of acupuncture for upper limb spastic hemiplegia following cerebral infarction, Li Hongpei et al. reported that acupuncture achieved faster MF improvement than CP after four weeks of treatment (4). These findings validate the superior efficacy of acupuncture in improving MF and highlight the influence of treatment duration on therapeutic outcomes. However, a major limitation was the lack of further subcategorization of acupuncture modalities. Ma et al. evaluated two acupuncture techniques for post-stroke upper limb spasticity with short-term treatment (≤1 month) (9). Post-intervention assessments showed that NM was significantly more effective than traditional acupuncture in improving MF. These findings are consistent with the present results, supporting its short-term efficacy. Nevertheless, substantial heterogeneity among novel techniques and insufficient subclassification in earlier studies limit the scope for detailed comparative analysis.

With respect to enhancement of physical capabilities, CP_BA_EA was identified as the most effective intervention for treatment durations exceeding one month, while CM_NM was superior for treatment durations of one month or less. Acupuncture stimulates muscles through a needling sensation, transmitting signals to deep spinal nerves that activate the central nervous system, thereby reestablishing connections with damaged neural synapses after stroke (10, 11). This mechanism facilitates active muscle contraction in affected limbs, repairs partially impaired nerves, and restores neural dominance, ultimately improving MF. EA further expands stimulation coverage, simultaneously activating motor and sensory nerves while enhancing the needling sensation of conventional acupuncture, thereby promoting muscle contraction and improving ligamentous tension. Clinical evidence indicates that this combined approach confers significant rehabilitative efficacy in paralysis recovery. The synergistic application of EA and BA thus enhances upper limb MF in the stroke population. In the subgroup analysis, CM_NM demonstrated favorable efficacy in improving MF among patients with treatment durations ≤1 month. Nevertheless, the statistical analysis was limited by a small sample size of only 30 patients. CM therapies encompass a wide range of modalities, including wax therapy, herbal fumigation and washing, and moxibustion. In the early treatment phase, these modalities possibly accelerate recovery of MF by unblocking meridians and promoting qi-blood circulation, thereby achieving superior outcomes during the initial period. As damaged nerves gradually regenerate during EA treatment, CP_BA_EA demonstrates increasing therapeutic advantages. Given the limited sample sizes of encompassed studies, these conclusions carry inherent limitations and should be interpreted with caution. Nonetheless, CM_NM exhibits considerable promise for future clinical application.

In the investigation of acupuncture modalities for alleviating post-stroke muscle hypertonia, Wang et al. reported that combined body and SA achieved significantly superior efficacy compared with BA alone in improving muscle tone and upper limb MF (12), a finding consistent with the results of the present NMA. CP_SA_BA demonstrated superior efficacy in reducing muscle cramp severity, yielding the most favorable therapeutic outcomes among the interventions studied. Contemporary medical theory attributes post-stroke spastic hypertonia in hemiplegic limbs to upper motor neuron lesions, which diminish inhibitory control over lower motor neurons (13). SA demonstrates neuroanatomical congruence with cortical somatotopy, enhances regional cerebral blood flow (rCBF) in corresponding sensorimotor cortices, and thereby accelerates tissue metabolism and neural reorganization. These mechanisms effectively alleviate spasticity through cortical neuroplasticity (14). BA enhances local circulation and tissue metabolism, alleviating muscle spasms. For example, Jingjin acupuncture (muscle-tendon region acupuncture) directly targets the muscular axis, inducing rapid relaxation and relieving muscle tension (15). CP applies isotonic contraction, repetitive stretching, and antagonistic muscle reversal techniques to strengthen antagonistic muscles and inhibit spasticity in hemiplegic limbs (16). Therefore, the integration of these three approaches, as exemplified by CP_SA_BA, demonstrates enhanced efficacy in alleviating muscle spasticity.

In addition, the role of acupuncture in enhancing ADL has been extensively investigated. Wang Xing’s research group conducted a meta-analysis evaluating the efficacy of acupuncture in improving the ADL index among post-stroke patients. The analysis demonstrated that EA is more effective than CP alone; BA combined with CP outperforms CP alone; and EA combined with CP yields superior results compared with CP alone (17). The foregoing findings suggest that acupuncture and acupuncture-related combination therapies may improve ADL in certain post-stroke populations. Zhang Yu conducted a systematic review assessing the efficacy of acupuncture for post-stroke limb spasticity (18). The review revealed that acupuncture surpasses other therapies in improving ADL. Collectively, these studies suggest that acupuncture enhances patients’ daily living abilities, although the optimal acupuncture modality for clinical application has not yet been conclusively established. For patients with a treatment duration of ≤1 month, CP_SA_BA was identified as the optimal regimen for improving daily MF, whereas combined physical therapy, BA, and EA (CP_BA_EA) emerged as the most effective strategy for patients with treatment durations exceeding 1 month. Improvement in ADL is closely linked to enhanced MF, and the combination of EA with BA possibly accelerates motor recovery, thereby laying a foundation for better performance in daily living activities. However, CP_BA_EA requires strong patient adherence and considerable family financial support. A subset of patients treated for ≤1 month may discontinue therapy or select more comfortable alternatives due to treatment-related discomfort. Among those who continue therapy, however, progressive improvement in ADL demonstrates notable clinical value throughout the course of treatment.

However, these results should be interpreted cautiously in the context of current international guidelines. The American Heart Association/American Stroke Association guidelines do not recommend acupuncture for improving ADL or MF (19). The National Institute for Health and Care Excellence guidelines do not recommend acupuncture or electroacupuncture for the routine management of post-stroke spasticity and instead highlight this area as a priority for further research (20). Therefore, although acupuncture and related combination therapies have not been widely endorsed in international guidelines, they may still serve as promising adjunctive interventions for post-stroke upper limb motor dysfunction.

This review incorporated a substantial number of RCTs and subcategorized various acupuncture modalities, thereby providing clearer evidence than previous literature regarding the therapeutic effects of distinct acupuncture types on post-stroke upper limb motor impairment. Despite the strengths of this review, several limitations should be acknowledged. First, key intervention details, such as acupoint selection, acupoint combinations, electroacupuncture parameters, and operator-dependent needling techniques, were inconsistently reported, increasing clinical heterogeneity and limiting comparability across studies. Second, some acupuncture modalities were supported by only a small number of trials, and their corresponding estimates and treatment rankings should therefore be interpreted with caution. Third, as an operator-dependent intervention, acupuncture presents inherent challenges to blinding, potentially introducing performance and detection bias. Fourth, long-term follow-up data, adverse event reporting, and patient-centered outcomes such as quality of life and satisfaction were limited, and treatment costs were not assessed; thus, it remains unclear whether short-term benefits translate into sustained functional recovery and broader clinical advantages. Fifth, variability in baseline characteristics, including stroke subtype, baseline MF, cognitive status, and comorbidities, may have affected the generalizability of the findings. Finally, publication bias cannot be excluded, and the predominance of studies conducted in Chinese populations may have led to an overestimation of treatment effects and may limit applicability to other healthcare settings.

## 5 Clinical recommendations

Based on the current network evidence and the prespecified certainty grading framework, acupuncture-related interventions should be considered adjunctive rather than stand-alone options in post-stroke upper limb rehabilitation. For treatment durations exceeding one month, CP_BA_EA may be considered for improving MF and ADL, whereas CP_SA_BA may be considered for reducing upper limb hypertonia. For treatment durations of one month or less, CM_NM may be considered for short-term MF improvement, CP_SA_BA for short-term enhancement of ADL, and CP_SA_BA for short-term relief of hypertonia. The certainty of these recommendations ranged from high to low, depending on the specific comparison and outcome, and all recommendations should be interpreted with caution, given the methodological limitations of the included evidence.

## 6 Conclusion

Based on the results of the NMA, CP_BA_EA demonstrated superior efficacy in improving MF in post-stroke hemiplegic patients, while combined moxibustion and neuromuscular therapy (CM_NM) emerged as a promising therapeutic option. For alleviation of post-stroke muscle hypertonia, combined physical therapy, SA, and BA (CP_SA_BA) showed significant benefits. Notably, in terms of enhancing ADL, CP_BA_EA exhibited substantial therapeutic potential for long-term treatment adherents. Acupuncture-related interventions may be incorporated as adjunctive components within comprehensive rehabilitation programs for post-stroke upper limb motor dysfunction. Nevertheless, in light of the cautious stance of major international guidelines and the methodological limitations of the available evidence, these therapies should currently be regarded as complementary rather than primary treatment strategies.

## Data Availability

The original contributions presented in the study are included in the article/Supplementary material, further inquiries can be directed to the corresponding author.
