# Supplementary material for: Systematic review and network meta-analysis of optimal acupuncture modalities for post-stroke upper limb motor dysfunction
Source: Front Neurol. 2026 Apr 20;17:1770511. doi: 10.3389/fneur.2026.1770511 (PMC13135975; doi:10.3389/fneur.2026.1770511)
Supplement: Supplementary file 1 [file Supplementary_file_1.docx]

**Supplementary File 1: Search strategies**

| **Database** | **Search details** | **Results** | **Time** | **language limits** |
| --- | --- | --- | --- | --- |
| **Pubmed** | #1((((((((((((Stroke[MeSH Terms]) OR (stroke*[Title/Abstract])) OR (Cerebrovascular Accident*[Title/Abstract])) OR (Brain Vascular Accident*[Title/Abstract])) OR (Apoplex*[Title/Abstract])) OR (CVA[Title/Abstract])) OR (CVAs[Title/Abstract])) OR (acute cerebrovascular lesion[Title/Abstract])) OR (brain accident[Title/Abstract])) OR (cerebral bleeding[Title/Abstract])) OR (poststroke[Title/Abstract])) OR (cerebral bleeding[Title/Abstract])) OR (poststroke[Title/Abstract]) | 398,396 | 23,Aug,2024 9:50:40 | NO |
|  | #2 (((((((((((((((((((Hemiplegia[MeSH Terms]) OR (Paralysis[MeSH Terms])) OR (Hemiplegia*[Title/Abstract])) OR (Paralys*[Title/Abstract])) OR (Palsy[Title/Abstract])) OR (Palsies[Title/Abstract])) OR (Plegia*[Title/Abstract])) OR (myoparalysis[Title/Abstract])) OR (myopleg*[Title/Abstract])) OR (paralytic disease[Title/Abstract])) OR (tardy apoplexy[Title/Abstract])) OR (hemiparalysis[Title/Abstract])) OR (dyskinesia[Title/Abstract])) OR (movement disorders[Title/Abstract])) OR (motor dysfunction[Title/Abstract])) OR (motor disturbance[Title/Abstract])) OR (motor function[Title/Abstract])) OR (movement function[Title/Abstract])) OR (activities of daily living[Title/Abstract])) OR (hemiparesis[Title/Abstract]) | 285,172 | 23,Aug,2024 9:54:38 |  |
|  | #3 (((((Upper Extremity[MeSH Terms]) OR (Upper Extremity[Title/Abstract])) OR (Upper Extremities[Title/Abstract])) OR (Upper Limb*[Title/Abstract])) OR (Membrum superius[Title/Abstract])) OR (hand function[Title/Abstract]) | 235,533 | 23,Aug,2024 9:56:39 |  |
|  | #4 (((((((((((((((((((((((((((((((Acupuncture[MeSH Terms]) OR (Electroacupuncture[MeSH Terms])) OR (Acupuncture[Title/Abstract])) OR (Electroacupuncture[Title/Abstract])) OR (Acupuncture*[Title/Abstract])) OR (Pharmacopuncture[Title/Abstract])) OR (Acupotom*[Title/Abstract])) OR (electroacupuncture[Title/Abstract])) OR (catgut embedding[Title/Abstract])) OR (shonishin[Title/Abstract])) OR (burnt needle therapy[Title/Abstract])) OR (fire needle therapy[Title/Abstract])) OR (fire needling[Title/Abstract])) OR (pharmaco-acupuncture[Title/Abstract])) OR (pharmaco-puncture[Title/Abstract])) OR (pharmacopuncture[Title/Abstract])) OR (electrical acupoint stimulation[Title/Abstract])) OR (electro-acupuncture[Title/Abstract])) OR (electroacupuncture[Title/Abstract])) OR (ACET[Title/Abstract])) OR (catgut implantation[Title/Abstract])) OR (catgut embedding[Title/Abstract])) OR (auriculotherapy[Title/Abstract])) OR (meridian*[Title/Abstract])) OR (acupoint*[Title/Abstract])) OR (warm needling[Title/Abstract])) OR (triple puncture[Title/Abstract])) OR (needle[Title/Abstract])) OR (manual-acupuncture[Title/Abstract])) OR (auriculo-acupuncture[Title/Abstract])) OR (auriculoacupuncture[Title/Abstract])) OR (dry needling[Title/Abstract]) | 171,024 | 23,Aug,2024 10:11:47 |  |
|  | #5 (((((((((randomized controlled trial[Title/Abstract]) OR (controlled clinical trial[Title/Abstract])) OR (randomized[Title/Abstract])) OR (placebo[Title/Abstract])) OR (randomly[Title/Abstract])) OR (trial[Title/Abstract])) OR (random*[Title/Abstract])) OR (placebo*[Title/Abstract])) OR (group*[Title/Abstract])) OR (control*[Title/Abstract]) | 896,9530 | 23,Aug,2024 10:22:16 |  |
|  | #6 #2 OR #3 | 503632 | 23,Aug,2024 10:36:54 |  |
|  | #7 #6 AND #1 AND #4 AND #5 | 538 | 23,Aug,2024 10:37:38 |  |
| **Cochrane Library** | #9 MeSH descriptor: [Upper Extremity] explode all trees | 10738 | 24,Aug,2024 22:17:37 | NO |
|  | #10 (Upper Extremity):ti,ab,kw OR (Upper Extremities):ti,ab,kw OR (Upper Limb*):ti,ab,kw OR (Membrum superius):ti,ab,kw OR (hand function):ti,ab,kw (Word variations have been searched) | 31266 |  |  |
|  | #11 #9 or #10 | 38155 |  |  |
|  | #12 MeSH descriptor: [Hemiplegia] explode all trees | 990 |  |  |
|  | #13 MeSH descriptor: [Paralysis] explode all trees | 2878 |  |  |
|  | #14 (Hemiplegia*):ti,ab,kw OR (Paralys*):ti,ab,kw OR (Palsy):ti,ab,kw OR (Palsies):ti,ab,kw OR (Plegia*):ti,ab,kw (Word variations have been searched) | 13124 |  |  |
|  | #15 (myoparalysis):ti,ab,kw OR (myopleg*):ti,ab,kw OR (paralytic disease):ti,ab,kw OR (tardy apoplexy):ti,ab,kw OR (hemiparalysis):ti,ab,kw (Word variations have been searched) | 198 |  |  |
|  | #16 (dyskinesia):ti,ab,kw OR (movement disorders):ti,ab,kw OR (motor dysfunction):ti,ab,kw OR (motor disturbance):ti,ab,kw OR (motor function):ti,ab,kw (Word variations have been searched) | 37576 |  |  |
|  | #17 (movement function):ti,ab,kw OR (activities of daily living):ti,ab,kw OR (hemiparesis):ti,ab,kw (Word variations have been searched) | 36712 |  |  |
|  | #18 #12 or #13 or #14 or #15 or #16 or #17 | 73143 |  |  |
|  | #19 MeSH descriptor: [Stroke] explode all trees | 17639 |  |  |
|  | #20 (stroke*):ti,ab,kw OR (Cerebrovascular Accident*):ti,ab,kw OR (Brain Vascular Accident*):ti,ab,kw OR (Apoplex*):ti,ab,kw OR (CVA):ti,ab,kw (Word variations have been searched) | 79056 |  |  |
|  | #21 (CVAs):ti,ab,kw OR (acute cerebrovascular lesion):ti,ab,kw OR (brain accident):ti,ab,kw OR (cerebral bleeding):ti,ab,kw OR (poststroke):ti,ab,kw (Word variations have been searched) | 12928 |  |  |
|  | #22 (brain insult*):ti,ab,kw OR (acute focal cerebral vasculopathy):ti,ab,kw OR (brain attack):ti,ab,kw OR (brain accident):ti,ab,kw OR (brain blood flow disturbance):ti,ab,kw (Word variations have been searched) | 6435 |  |  |
|  | #23 (cerebral apoplexia):ti,ab,kw OR (cerebral insult):ti,ab,kw OR (cerebral vascular accident):ti,ab,kw OR (cerebral vascular insufficiency):ti,ab,kw OR (cerebro vascular accident):ti,ab,kw (Word variations have been searched) | 677 |  |  |
|  | #24 (cerebrovascular arrest):ti,ab,kw OR (cerebrovascular failure):ti,ab,kw OR (cerebrovascular injury):ti,ab,kw OR (cerebrovascular insufficiency):ti,ab,kw OR (cerebrovascular insult):ti,ab,kw (Word variations have been searched) | 6361 |  |  |
|  | #25 (cerebrum vascular accident):ti,ab,kw OR (insultus cerebralis):ti,ab,kw OR (ischaemic seizure):ti,ab,kw OR (ischemic seizure):ti,ab,kw (Word variations have been searched) | 418 |  |  |
|  | #26 #19 or #20 or #21 or #22 or #23 or #24 or #25 | 83273 |  |  |
|  | #27 MeSH descriptor: [Acupuncture] explode all trees | 224 |  |  |
|  | #28 MeSH descriptor: [Electroacupuncture] explode all trees | 1175 |  |  |
|  | #29 MeSH descriptor: [Acupuncture, Ear] explode all trees | 266 |  |  |
|  | #30 MeSH descriptor: [Acupuncture Therapy] explode all trees | 7142 |  |  |
|  | #31 (Acupuncture*):ti,ab,kw OR (Pharmacopuncture):ti,ab,kw OR (Acupotom*):ti,ab,kw OR (electroacupuncture):ti,ab,kw OR (catgut embedding):ti,ab,kw (Word variations have been searched) | 21874 |  |  |
|  | #32 (shonishin):ti,ab,kw OR (burnt needle therapy):ti,ab,kw OR (fire needle therapy):ti,ab,kw OR (fire needling):ti,ab,kw OR (pharmaco-acupuncture):ti,ab,kw (Word variations have been searched) | 328 |  |  |
|  | #33 (pharmaco-puncture):ti,ab,kw OR (pharmacopuncture):ti,ab,kw OR (electrical acupoint stimulation):ti,ab,kw OR (electro-acupuncture):ti,ab,kw OR (ACET):ti,ab,kw (Word variations have been searched) | 17183 |  |  |
|  | #34 (catgut implantation):ti,ab,kw OR (catgut embedding):ti,ab,kw OR (auriculotherapy):ti,ab,kw OR (meridian*):ti,ab,kw OR (acupoint*):ti,ab,kw (Word variations have been searched) | 7445 |  |  |
|  | #35 (warm needling):ti,ab,kw OR (triple puncture):ti,ab,kw OR (needle):ti,ab,kw OR (manual-acupuncture):ti,ab,kw OR (auriculo-acupuncture):ti,ab,kw (Word variations have been searched) | 23753 |  |  |
|  | #36 (auriculoacupuncture):ti,ab,kw OR (dry needling):ti,ab,kw (Word variations have been searched) | 1637 |  |  |
|  | #37 #27 or #28 or #29 or #30 or #31 or #32 or #33 or #34 or #35 or #36 | 58909 |  |  |
|  | #38 (randomized controlled trial):ti,ab,kw OR (controlled clinical trial):ti,ab,kw OR (randomized):ti,ab,kw OR (placebo):ti,ab,kw OR (randomly):ti,ab,kw (Word variations have been searched) | 1518011 |  |  |
|  | #39 (trial):ti,ab,kw OR (random*):ti,ab,kw OR (placebo*):ti,ab,kw OR (group*):ti,ab,kw OR (control*):ti,ab,kw (Word variations have been searched) | 1821771 |  |  |
|  | #40 #38 or #39 | 1821771 |  |  |
|  | #41 #11 or #18 | 99326 |  |  |
|  | #42 #41 and #26 and #37 and #40 | 985 |  |  |
| **Web of Science** | #1 TS=(Upper Extremity OR Membrum superius OR Upper Extremities OR Upper Limb* OR hand function) | 219794 | 30,Aug, 2024 09:30:40 | NO |
|  | #2 TS=(Hemiplegia* OR Paralys* OR Palsy OR Palsies OR Plegia* OR myoparalysis OR myopleg* OR paralytic disease OR tardy apoplexy OR hemiparalysis OR dyskinesia OR movement disorders OR motor dysfunction OR motor disturbance OR motor function OR activities of daily living OR hemiparesis) | 460878 | 30,Aug, 2024  09:30:54 |  |
|  | #3 TS=(stroke* OR Cerebrovascular Accident* OR Brain Vascular Accident* OR Apoplex* OR CVA OR CVAs OR acute cerebrovascular lesion OR brain accident OR cerebral bleeding OR poststroke OR brain insult* OR acute focal cerebral vasculopathy OR brain attack OR brain blood flow disturbance OR cerebral apoplexia OR cerebral insult OR cerebral vascular accident OR cerebral vascular insufficiency OR cerebro vascular accident OR cerebrovascular arrest OR cerebrovascular failure OR cerebrovascular injury OR cerebrovascular insufficiency OR cerebrovascular insult OR cerebrum vascular accident OR insultus cerebralis OR ischaemic seizure OR ischemic seizure) | 550831 | 30,Aug, 2024 09:31:02 |  |
|  | #4 TS=(Acupuncture* OR Pharmacopuncture OR Acupotom* OR electroacupuncture OR catgut embedding OR shonishin OR burnt needle therapy OR fire needle therapy OR fire needling OR pharmaco-acupuncture OR pharmaco-puncture OR pharmacopuncture OR electrical acupoint stimulation OR electro-acupuncture OR ACET OR catgut implantation OR catgut embedding OR auriculotherapy OR meridian* OR acupoint* OR warm needling OR triple puncture OR needle OR manual-acupuncture OR auriculo-acupuncture OR auriculoacupuncture OR dry needling) | 245578 | 30,Aug, 2024  09:31:17 |  |
|  | #5 TS=(randomized controlled trial OR controlled clinical trial OR randomized OR placebo OR randomly OR trial OR random* OR placebo* OR group* OR control*) | 16121830 | 30,Aug, 2024  09:31:23 |  |
|  | #6 #1 OR #2 | 651110 | 30,Aug, 2024  09:31:31 |  |
|  | #7 #3 AND #4 AND #5 AND #6 | 496 | 30,Aug, 2024  09:31:38 |  |
| **Embase** | #1 'upper limb'/exp | 400,174 | 24,Aug, 2024 | NO |
|  | #2 'upper limb':ab,ti OR 'upper extremity':ab,ti OR 'upper extremities':ab,ti OR 'upper limb*':ab,ti OR 'membrum superius':ab,ti OR 'hand function':ab,ti | 100,299 | 24,Aug, 2024 |  |
|  | #3 #1 OR #2 | 442,643 | 24,Aug, 2024 |  |
|  | #4 'paralysis'/exp | 411,581 | 24,Aug, 2024 |  |
|  | #5 'hemiplegia'/exp | 24,962 | 24,Aug, 2024 |  |
|  | #6 hemiplegia*:ab,ti OR paralys*:ab,ti OR palsy:ab,ti OR palsies:ab,ti OR plegia*:ab,ti OR myoparalysis:ab,ti OR myopleg*:ab,ti OR 'paralytic disease':ab,ti OR 'tardy apoplexy':ab,ti OR hemiparalysis:ab,ti OR paralysis:ab,ti OR hemiplegia:ab,ti OR dyskinesia:ab,ti OR 'movement disorders':ab,ti OR 'motor dysfunction':ab,ti OR 'motor disturbance':ab,ti OR 'motor function':ab,ti OR 'movement function':ab,ti OR 'activities of daily living':ab,ti OR hemiparesis:ab,ti | 318,543 | 24,Aug, 2024 |  |
|  | #7 #4 OR #5 OR #6 | 579,890 | 24,Aug, 2024 |  |
|  | #8 #3 OR #7 | 986,195 | 24,Aug, 2024 |  |
|  | #9 'cerebrovascular accident'/exp | 465,094 | 24,Aug, 2024 |  |
|  | #10 'cerebrovascular accident*':ab,ti OR stroke*:ab,ti OR 'brain vascular accident*':ab,ti OR apoplex*:ab,ti OR cva:ab,ti OR cvas:ab,ti OR 'acute cerebrovascular lesion':ab,ti OR 'cerebral bleeding':ab,ti OR poststroke:ab,ti OR 'brain insult*':ab,ti OR 'acute focal cerebral vasculopathy':ab,ti OR 'brain attack':ab,ti OR 'brain accident':ab,ti OR 'brain blood flow disturbance':ab,ti OR 'cerebral apoplexia':ab,ti OR 'cerebral insult':ab,ti OR 'cerebral vascular accident':ab,ti OR 'cerebral vascular insufficiency':ab,ti OR 'cerebro vascular accident':ab,ti OR 'cerebrovascular arrest':ab,ti OR 'cerebrovascular failure':ab,ti OR 'cerebrovascular injury':ab,ti OR 'cerebrovascular insufficiency':ab,ti OR 'cerebrovascular insult':ab,ti OR 'cerebrum vascular accident':ab,ti OR 'insultus cerebralis':ab,ti OR 'ischaemic seizure':ab,ti OR 'ischemic seizure':ab,ti | 561,228 | 24,Aug, 2024 |  |
|  | #11 #9 OR #10 | 675,792 | 24,Aug, 2024 |  |
|  | #12 'acupuncture'/exp | 60,806 | 24,Aug, 2024 |  |
|  | #13 'warm acupuncture'/exp | 260 | 24,Aug, 2024 |  |
|  | #14 'electroacupuncture'/exp | 10,132 | 24,Aug, 2024 |  |
|  | #15 'catgut embedding'/exp | 227 | 24,Aug, 2024 |  |
|  | #16 'pharmacopuncture'/exp | 206 | 24,Aug, 2024 |  |
|  | #17 'acupuncture analgesia'/exp | 1,876 | 24,Aug, 2024 |  |
|  | #18. 'auricular acupuncture'/exp | 954 | 24,Aug, 2024 |  |
|  | #19 acupuncture*:ab,ti OR acupotom*:ab,ti OR electroacupuncture:ab,ti OR shonishin:ab,ti OR 'burnt needle therapy':ab,ti OR 'fire needle therapy':ab,ti OR 'fire needling':ab,ti OR 'pharmaco acupuncture':ab,ti OR 'pharmaco puncture':ab,ti OR pharmacopuncture:ab,ti OR 'electrical acupoint stimulation':ab,ti OR 'electro acupuncture':ab,ti OR acet:ab,ti OR'catgut implantation':ab,ti OR 'catgut embedding':ab,ti OR auriculotherapy:ab,ti OR meridian*:ab,ti OR acupoint*:ab,ti OR 'warm needling':ab,ti OR 'triple puncture':ab,ti OR needle:ab,ti OR 'manual acupuncture':ab,ti OR 'auriculo acupuncture':ab,ti OR auriculoacupuncture:ab,ti OR 'dry needling':ab,ti | 237,295 | 24,Aug, 2024 |  |
|  | #20 #12 OR #13 OR #14 OR #15 OR #16 OR #17 OR #18 OR #19 | 253,034 | 24,Aug, 2024 |  |
|  | #21 'randomized controlled trial':ab,ti OR 'controlled clinical trial':ab,ti OR randomized:ab,ti OR placebo:ab,ti OR randomly:ab,ti OR trial:ab,ti OR random*:ab,ti OR placebo*:ab,ti OR group*:ab,ti OR control*:ab,ti | 11,967,498 | 24,Aug, 2024 |  |
|  | #22 #8 AND #11 AND #20 AND #21 | 754 | 24,Aug, 2024 |  |
| **SinoMed** | #1 "上肢"[不加权:扩展] |  |  | NO |
|  | #2 "麻痹"[不加权:扩展] |  |  |  |
|  | #3 "偏瘫"[不加权:扩展] |  |  |  |
|  | #4 "麻痹OR偏瘫"[不加权:扩展] |  |  |  |
|  | #5 #1 OR #4 |  |  |  |
|  | #6 "卒中"[不加权:扩展] |  |  |  |
|  | #7 "针灸疗法"[不加权:扩展] |  |  |  |
|  | #8 "温针疗法OR耳廓针刺术OR耳埋OR针灸OR温针灸OR电针OR针刺OR 耳针 OR 头针 " [常用字段:智能] |  |  |  |
|  | #9 "头皮针 OR温和灸" [常用字段:智能] |  |  |  |
|  | #10 #7 OR #8 OR #9 |  |  |  |
|  | #11 "随机 OR RCT" [常用字段:智能] |  |  |  |
|  | #12 #5 AND #6 AND #10 AND #11 | 751 | 28,Aug, 2024 |  |
| **China National Knowledge Infrastructure** | TKA = ('针灸'+'温针灸'+'电针'+'针刺'+'耳针'+'头针'+'头皮针'+'温针疗法'+'耳廓针刺术'+'耳埋'+'体针'+'腹针'+'眼针'+'腕踝针') AND TKA = ('卒中'+'脑缺血'+'脑梗死'+'脑出血'+'中风'+'脑血管意外') AND TKA = ('运动功能障碍'+'麻痹'+'瘫痪'+'单瘫'+'偏瘫'+'半身不遂'+'偏枯'+'偏风') AND TKA = ('随机'+'RCT') AND TKA = '上肢' | 764 | 29,Aug, 2024 | NO |
| **Wanfang** | 卒中 OR 脑缺血 OR 脑梗死 OR 脑出血 OR 中风 OR 脑血管意外) and 摘要:(针灸 OR 温针灸 OR 电针 OR 针刺 OR 耳针 OR 头针 OR 头皮针OR 温针疗法 OR 耳廓针刺术 OR 耳埋 OR 体针 OR 腹针 OR 眼针 OR 腕踝针) and 摘要:(运动功能障碍 OR 麻痹 OR 瘫痪 OR 单瘫 OR 偏瘫 OR 半身不遂 OR 偏枯 OR 偏风 ) and 摘要:(上肢) and 摘要:(随机 OR RCT) | 764 | 29,Aug, 2024 | NO |
| **China Science and Technology Journal Database** | (((((((((((((((((((((((((((((((((摘要=卒中 OR 摘要=脑缺血) OR 摘要=脑梗死) OR 摘要=脑出血) OR 摘要=中风) OR 摘要=脑血管意外) AND (((((((((((((摘要=针灸 OR 摘要=温针灸) OR 摘要=电针) OR 摘要=针刺) OR 摘要=耳针) OR 摘要=头针) OR 摘要=头皮针) OR 摘要=温针疗法) OR 摘要=耳廓针刺术) OR 摘要=耳埋) OR 摘要=体针) OR 摘要=腹针) OR 摘要=眼针) OR 摘要=腕踝针)))))))) AND (((((((摘要=运动功能障碍 OR 摘要=麻痹) OR 摘要=瘫痪) OR 摘要=单瘫) OR 摘要=偏瘫) OR 摘要=半身不遂) OR 摘要=偏枯) OR 摘要=偏风)))))))) AND 摘要=上肢))))))) AND (摘要=随机 OR 摘要=RCT)))))))) | 588 | 28,Aug, 2024 | NO |


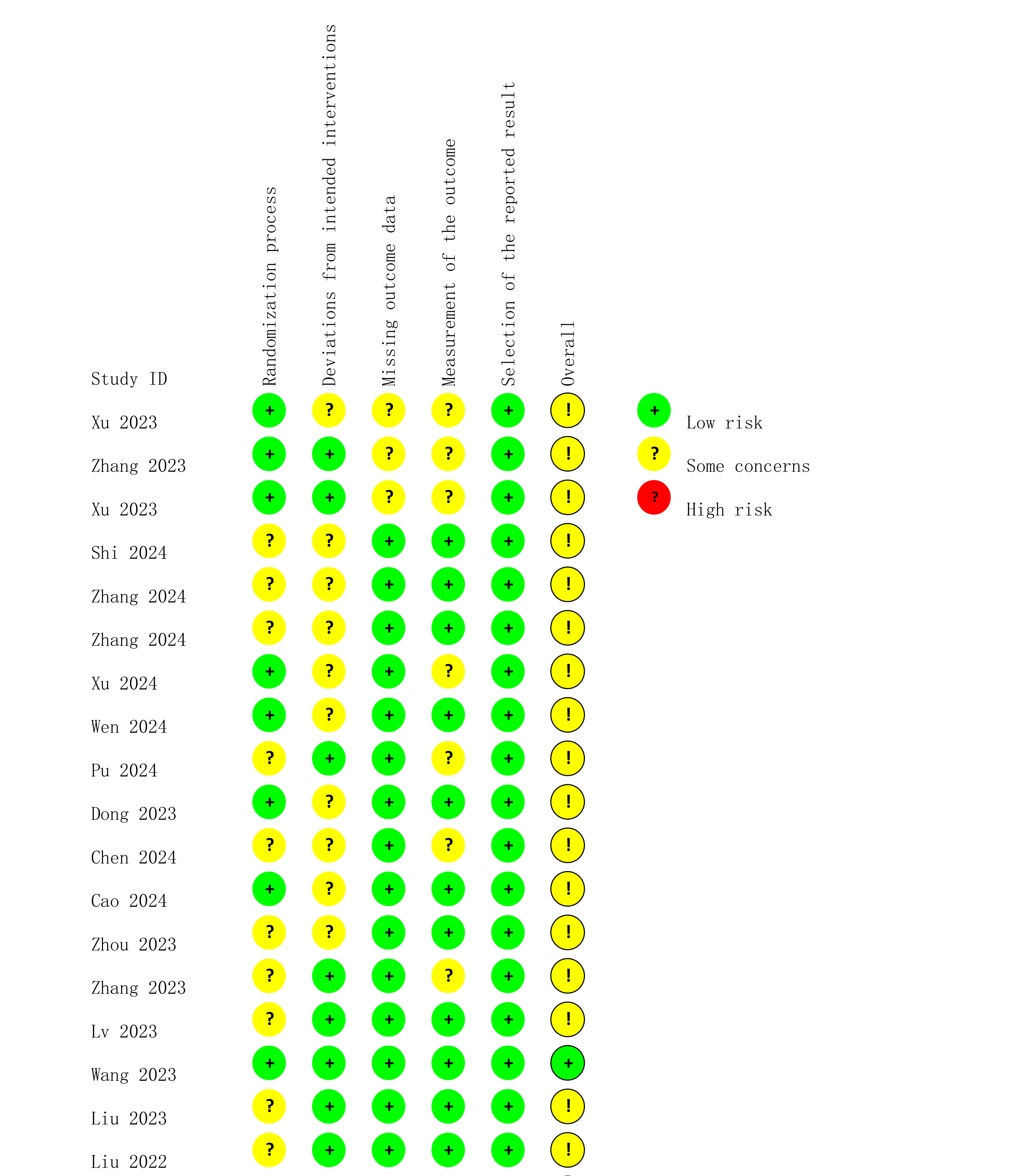


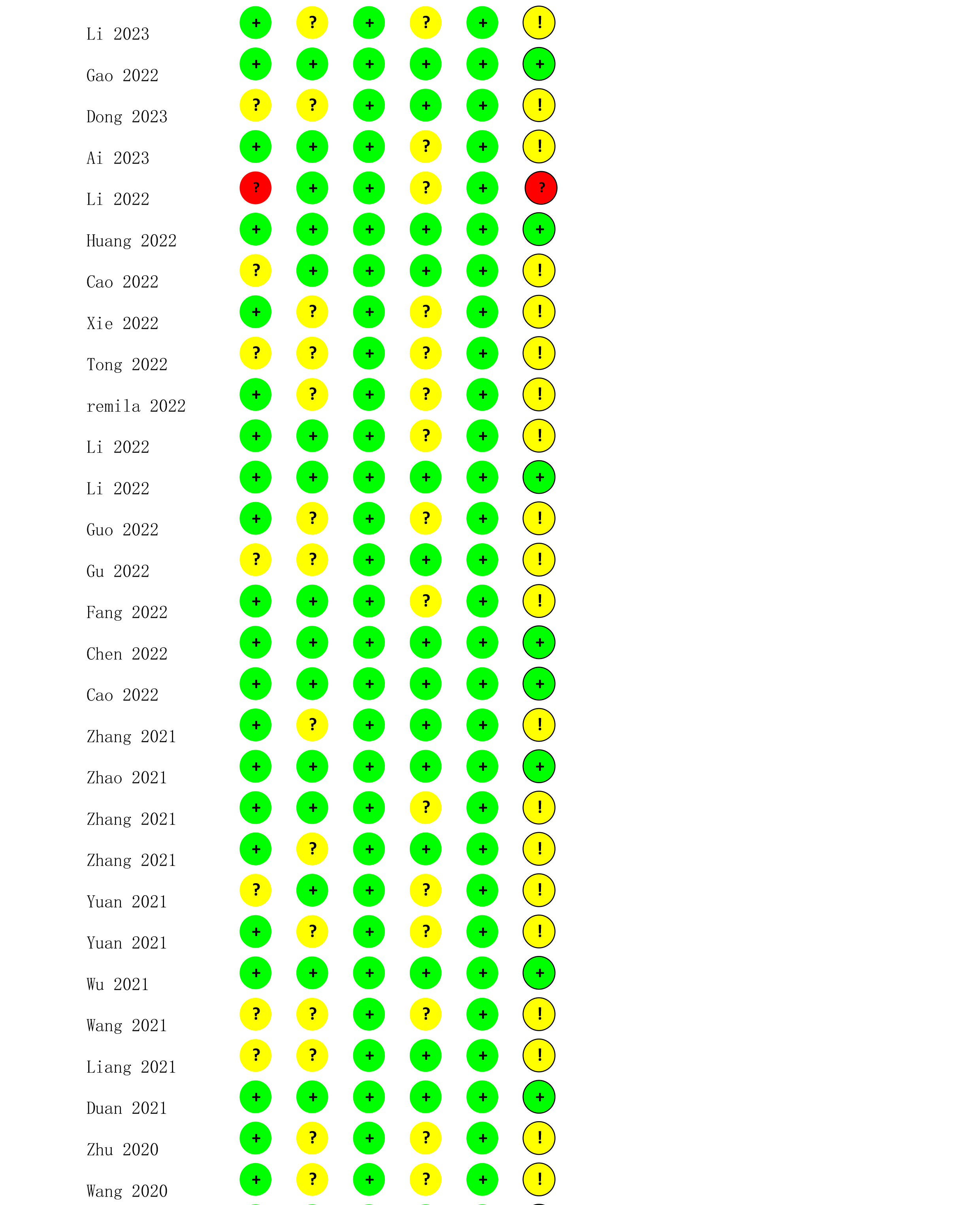


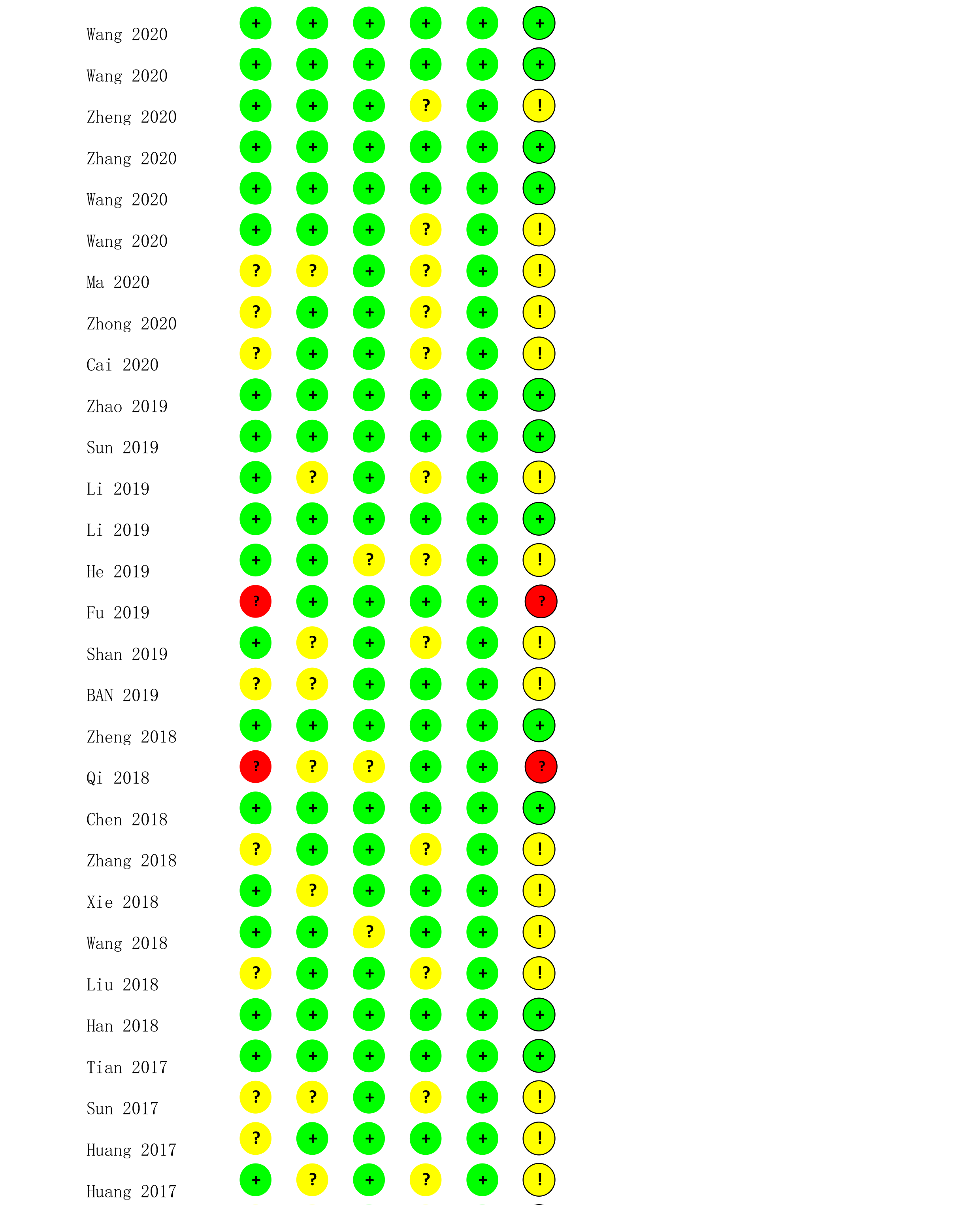


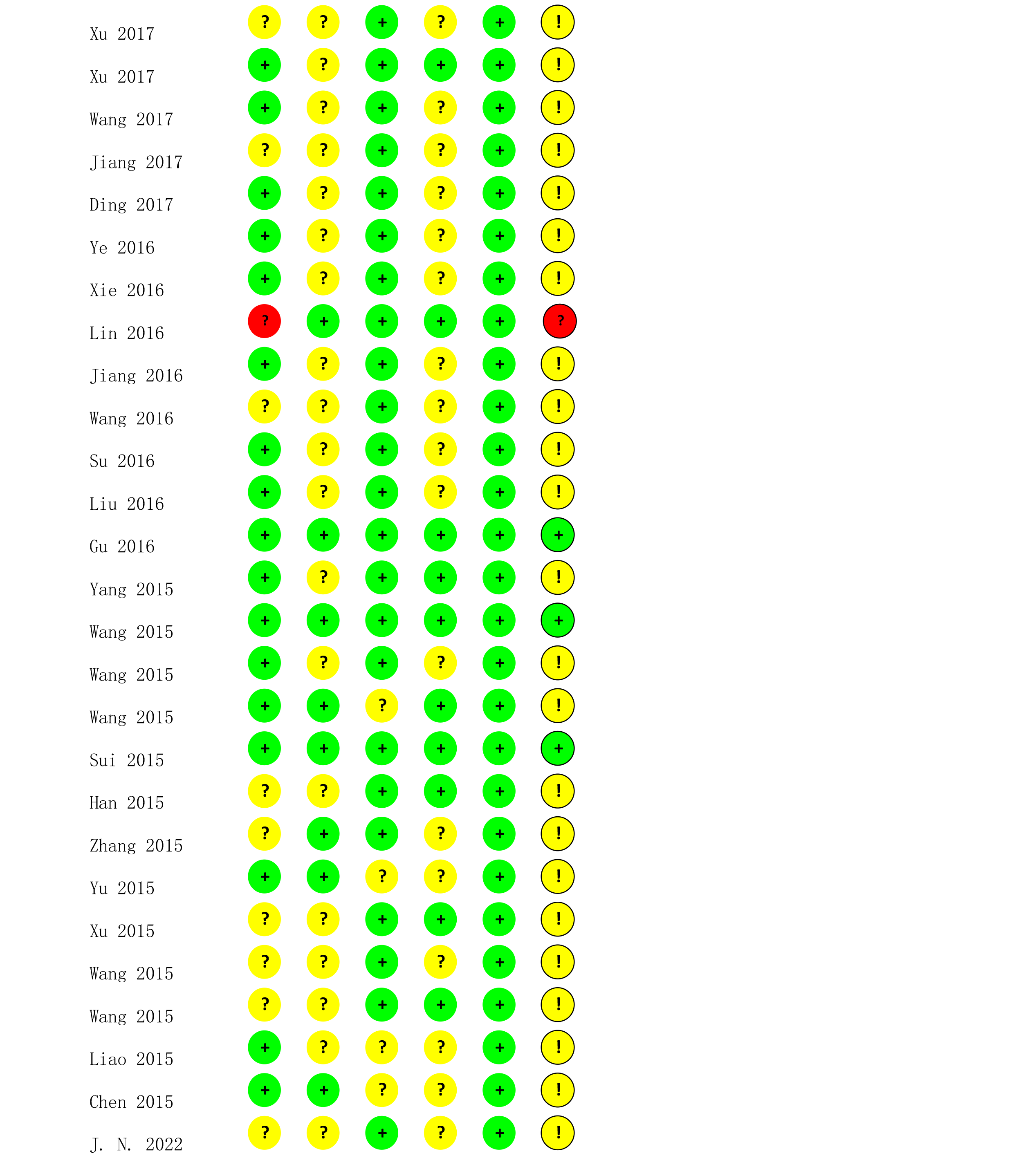


**Figure S1** Risk of bias assessment for included studies.

**Table S1.** Cumulative probability statistics.

| Outcomes | BA | CP | EA | SA | SH | WA | FN | PM | CM | WM | NM | CP_BA | CP_EA | CP_SA | CP_NM | CP_WA | CP_WAN | SA_BA | SA_NM | BA_PM | BA_EA | BA_WM | CM_BA | CM_FN | CM_EA | CM_NM | CP_BA_EA | CP_BA_PM | CP_CM_BA | CP_NM_PM | CP_NM_EA | CP_SA_BA | CP_SA_PM | CM_SA_BA | CM_BA_EA | CM_BA_PM | SA_BA_EA | CP_SA_BA_EA | CP_CM_SA_BA |
| --- | --- | --- | --- | --- | --- | --- | --- | --- | --- | --- | --- | --- | --- | --- | --- | --- | --- | --- | --- | --- | --- | --- | --- | --- | --- | --- | --- | --- | --- | --- | --- | --- | --- | --- | --- | --- | --- | --- | --- |
| Activity of daily living(＞1month) | 32.13% | 13.62% | 15.77% | 13.60% | 2.86% | 77.22% | / | / | / | / | / | 59.74% | 38.50% | 71.37% | 67.49% | 41.86% | / | 22.32% | / | / | / | / | / | / | / | / | 99.13% | / | 70.08% | 27.82% | / | 88.67% | 71.42% | / | / | / | / | / | / |
| Activity of daily living(≤1month) | 17.53% | 36.95% | 27.06% | 8.03% | 18.35% | 63.71% | / | 21.79% | / | / | 17.15% | 57.14% | 42.88% | 51.26% | 60.0% | 48.37% | 74.85% | 64.73% | / | 43.34% | / | / | 34.54% | / | / | / | 75.79% | 33.94% | 74.62% | / | 61.03% | 96.43% | / | / | / | / | / | 47.27% | 76.34% |
| Motor function(＞1month) | 31.46% | 18.09% | / | / | 64.27% | 59.20% | / | / | / | / | / | 63.17% | 30.29% | 70.67% | 76.79% | 31.17% | / | 7.94% | 36.92% | / | / | / | / | / | / | / | 79.67% | / | 56.42% | 74.79% | / | 72.81% | 59.01% | 27.94% | / | / | 37.03% | 52.35% | / |
| Motor function(≤1month) | 18.33% | 21.19% | 18.60% | 3.63% | 17.54% | 46.45% | 44.01% | 8.41% | 40.28% | 47.81% | 9.21% | 49.42% | 42.06% | 47.02% | 55.16% | 27.93% | 70.43% | 48.23% | / | 68.52% | 89.80% | 76.78% | 52.56% | 72.76% | 49.52% | 99.96% | 76.52% | 59.99% | 68.32% | / | 35.88% | 86.41% | / | / | 95.21% | 86.13% | / | / | 15.93% |
| Muscle cramp severity(＞1month) | 37.52% | 23.75% | / | / | / | / | / | / | / | / | / | 36.47% | / | 55.48% | 41.32% | / | / | 29.19% | / | / | / | / | / | / | / | / | / | / | 77.80% | / | / | 81.53% | / | 66.94% | / | / | / | / | / |
| Muscle cramp severity(≤1month) | 22.65% | 23.56% | / | 24.84% | / | / | 68.67% | 23.22% | 71.29% | 42.25% | / | 40.57% | / | 52.04% | 48.47% | / | / | 52.59% | / | 47.96% | / | / | 56.20% | 57.38% | / | / | / | 66.24% | / | / | 46.30% | 87.77% | / | / | / | / | / | / | / |
| Visual Analogue Scale (VAS) | / | 9.90% | / | / | / | / | / | / | / | / | / | 44.88% | 46.64% | / | / | / | 62.47% | / | / | / | / | / | / | / | / | / | / | / | 66.87% | / | / | 69.24% | / | / | / | / | / | / | / |
| Clinical response rate | 14.47% | 6.80% | 3.79% | / | / | / | 71.89% | / | 78.77% | / | / | 41.49% | 28.16% |  | 50.72% |  | 58.79% | 41.97% | / | / | / | / | 31.98% | 88.11% | 77.89% | 43.65% | 59.59% | / | 70.11% | / | / | 81.54% | / | / | / | / | / | / | / |
| Progression rate to Grade 0 on the Modified Ashworth Scale(MAS) | 33.51% | 21.82% | / | / | / | 41.31% | 72.92% | / | / | / | / | 72.77% | 41.56% | 41.36% | / | 41.92% | / | / | / | / | / | / | / | / | / | / | 40.09% | / | 47.85% | / | / | / | / | 86.90% | / | / | / | / | / |
| Spasm relief effective rate | 75.66% | 21.76% | / | 51.17% | 22.78% | / | / | / | / | / | / | 70.37% | / | / | / | / | / | / | / | / | / | / | / | / | / | / | / | / | 56.71% | / | / | 51.55% | / | / | / | / | / | / | / |

**Table S2.** Local inconsistency check

| Outcomes | Comparison | Direct | Indirect | Network | P.value CrI |
| --- | --- | --- | --- | --- | --- |
| Activity of daily living(＞1month) | CP BA | -0.44 | -0.32 | -0.38 | 0.75 |
|  | CP_BA BA | 0.69 | 0.57 | 0.65 | 0.75 |
|  | CP_BA CP | 1.0 | 1.12 | 1.03 | 0.75 |
|  | CP_EA CP | 0.50 | 0.68 | 0.52 | 0.78 |
|  | CP_SA_BA CP | 1.76 | 1.71 | 1.75 | 0.94 |
|  | CP_WA CP | 0.62 | 0.62 | 0.61 | 0.999 |
|  | EA CP | 0.08 | -0.10 | 0.004 | 0.77 |
|  | SA_BA CP | 0.14 | 0.19 | 0.16 | 0.94 |
|  | WA CP | 1.44 | 1.44 | 1.44 | 0.999 |
|  | EA CP_EA | -0.60 | -0.42 | -0.52 | 0.78 |
|  | SA_BA CP_SA_BA | -1.57 | -1.62 | -1.59 | 0.94 |
|  | WA CP_WA | 0.83 | 0.83 | 0.83 | 0.99 |
| Activity of daily living(≤1month) | BA_PM BA | 0.91 | 0.90 | 0.91 | 0.9998 |
|  | CP BA | 0.10 | 1.07 | 0.81 | 0.44 |
|  | CP_BA BA | 0.71 | 1.78 | 1.43 | 0.42 |
|  | EA BA | 0.04 | 0.57 | 0.34 | 0.71 |
|  | PM BA | 0.02 | 0.008 | 0.21 | 0.996 |
|  | SA_BA BA | 5.43 | -0.04 | 1.68 | ＜0.0001 |
|  | SH BA | -0.60 | 0.84 | 0.21 | 0.20 |
|  | PM BA_PM | -0.89 | -0.88 | -0.89 | 0.995 |
|  | CP_BA CP | 1.1 | 0.005 | 0.62 | 0.18 |
|  | CP_BA_EA CP | 1.3 | 1.22 | 1.28 | 0.94 |
|  | CP_CM_BA CP | 0.85 | 1.77 | 1.24 | 0.43 |
|  | CP_EA CP | 0.42 | -0.54 | 0.16 | 0.49 |
|  | CP_SA CP | 0.74 | -0.48 | 0.42 | 0.4 |
|  | CP_SA_BA CP | 2.34 | 3.47 | 2.65 | 0.29 |
|  | CP_WA CP | 0.34 | 0.35 | 0.34 | 0.9997 |
|  | CP_WAN CP | 0.32 | 2.53 | 1.33 | 0.16 |
|  | EA CP | -0.06 | -0.78 | -0.47 | 0.62 |
|  | SA_BA CP | -0.05 | 1.66 | 0.87 | 0.09 |
|  | SH CP | -0.3 | -1 | -0.79 | 0.57 |
|  | WA CP | 0.90 | 0.88 | 0.9 | 0.995 |
|  | CP_BA_EA CP_BA | 0.02 | 0.93 | 0.66 | 0.48 |
|  | CP_CM_BA CP_BA | 1.02 | 0.10 | 0.62 | 0.43 |
|  | CP_WAN CP_BA | 1.74 | -0.46 | 0.71 | 0.15 |
|  | CP_EA CP_BA_EA | -1.67 | -0.71 | -1.13 | 0.5 |
|  | SH CP_SA | -0.56 | -1.77 | -1.21 | 0.41 |
|  | SA_BA CP_SA_BA | -2.16 | -1.04 | -1.77 | 0.29 |
|  | WA CP_WA | 0.55 | 0.55 | 0.55 | 0.998 |
|  | SH EA | -0.24 | -0.39 | -0.32 | 0.9 |
| Motor Function(＞1month) | CP BA | -0.43 | -0.36 | -0.39 | 0.92 |
|  | CP_BA BA | 0.88 | 0.81 | 0.86 | 0.92 |
|  | CP_BA CP | 1.36 | 0.85 | 1.24 | 0.44 |
|  | CP_NM CP | 0.7 | 2.75 | 1.69 | 0.09 |
|  | CP_SA_BA CP | 1.32 | 3.01 | 1.5 | 0.19 |
|  | CP_WA CP | 0.32 | 0.32 | 0.32 | 0.999 |
|  | SA_BA CP | 0.13 | -1.57 | -0.61 | 0.19 |
|  | WP CP | 1.14 | 1.15 | 1.15 | 0.996 |
|  | CP_NM CP_BA | 1.41 | -0.65 | 0.44 | 0.09 |
|  | SA_BA CP_SA_BA | -2.89 | -1.2 | -2.12 | 0.19 |
|  | WA CP_WA | 0.83 | 0.83 | 0.82 | ＞0.9999 |
| Motor Function(≤1month) | BA_PM BA | 1.13 | 2.32 | 1.6 | 0.29 |
|  | CM_BA BA | 0.93 | 1.28 | 1.05 | 0.77 |
|  | CP BA | -0.58 | 0.57 | 0.1 | 0.02 |
|  | CP_BA BA | 0.61 | 1.11 | 0.94 | 0.42 |
|  | EA BA | 0.06 | -0.001 | 0.012 | 0.94 |
|  | FN BA | 1.16 | -0.05 | 0.78 | 0.18 |
|  | PM BA | -0.2 | -1.38 | -0.65 | 0.3 |
|  | SA_BA BA | 3.17 | 0.14 | 0.9 | 0.00034 |
|  | SH BA | 0.03 | -0.13 | -0.05 | 0.81 |
|  | PM BA_PM | -2.52 | -1.33 | -2.26 | 0.29 |
|  | CM_EA CM | 0.53 | -0.01 | 0.26 | 0.6 |
|  | CM_FN CM | 0.89 | 1.24 | 1.05 | 0.73 |
|  | EA CM | -0.3 | -0.9 | -0.066 | 0.52 |
|  | FN CM | -0.36 | 0.47 | 0.11 | 0.38 |
|  | CM_FN CM_BA | 0.56 | 0.91 | 0.68 | 0.77 |
|  | EA CM_EA | -0.79 | -1.34 | -0.93 | 0.6 |
|  | FN CM_FN | -1.24 | -0.64 | -0.95 | 0.56 |
|  | CP_BA CP | 1 | 0.49 | 0.83 | 0.23 |
|  | CP_BA_EA CP | 1.75 | 1.79 | 1.76 | 0.97 |
|  | CP_CM_BA CP | 1.24 | 1.87 | 1.4 | 0.32 |
|  | CP_EA CP | 0.61 | 0.67 | 0.63 | 0.91 |
|  | CP_SA CP | 0.9 | 0.23 | 0.76 | 0.46 |
|  | CP_SA_BA CP | 2.18 | 2.98 | 2.34 | 0.22 |
|  | CP_WA CP | 0.17 | 0.17 | 0.17 | 0.997 |
|  | CP_WAN CP | 0.7 | 2.49 | 1.55 | 0.08 |
|  | EA CP | -0.06 | -0.11 | -0.09 | 0.94 |
|  | SA_BA CP | 0.31 | 1.17 | 0.8 | 0.19 |
|  | SH CP | -0.46 | -0.05 | -0.15 | 0.62 |
|  | WA CP | 0.75 | 0.75 | 0.75 | 0.998 |
|  | CP_BA_EA CP_BA | 0.05 | 1.3 | 0.93 | 0.14 |
|  | CP_CM_BA CP_BA | 0.96 | 0.33 | 0.57 | 0.32 |
|  | CP_WAN CP_BA | 1.58 | -0.2 | 0.71 | 0.08 |
|  | CP_EA CP_BA_EA | -2.02 | -0.67 | -1.13 | 0.12 |
|  | EA CP_EA | -1.05 | -0.27 | -0.72 | 0.21 |
|  | SH CP_SA | -0.5 | -1.17 | -0.92 | 0.46 |
|  | SA_BA CP_SA_BA | -1.86 | -1.05 | -1.54 | 0.22 |
|  | WA CP_WA | 0.58 | 0.58 | 0.58 | 0.99 |
|  | SH EA | -0.32 | 0.06 | -0.06 | 0.65 |
| Muscle cramp severity(＞1month) | CP BA | 0.3 | 0.30 | 0.30 | 0.995 |
|  | CP_BA BA | 0.03 | 0.03 | 0.03 | 0.997 |
|  | CP_BA CP | -0.27 | -0.28 | -0.28 | 0.997 |
|  | CP_SA_BA CP | -1.86 | -1.86 | -1.86 | 0.999 |
|  | SA_BA CP | -0.09 | -0.09 | -0.09 | 0.998 |
|  | SA_BA CP_SA_BA | 1.78 | 1.78 | 1.77 | 0.999 |
| Muscle cramp severity(≤1month) | BA_PM BA | -0.84 | -0.85 | -0.85 | 0.999 |
|  | CP BA | 1.28 | -2.42 | -0.07 | 0.01 |
|  | PM BA | 0.11 | 0.11 | 0.12 | 0.999 |
|  | SA_BA BA | -2.47 | 1.23 | -1.02 | 0.01 |
|  | PM BA_PM | 0.96 | 0.96 | 0.96 | 0.9999 |
|  | CM_FN CM | -0.08 | -0.09 | -0.08 | 0.999 |
|  | FN CM | 0.11 | 0.10 | 0.11 | 0.999 |
|  | FN CM_FN | 0.20 | 0.20 | 0.20 | 0.999 |
|  | CP_SA_BA CP | -2.24 | -3.44 | -2.69 | 0.50 |
|  | SA_BA CP | -0.06 | -1.89 | -0.96 | 0.22 |
|  | SA_BA CP_SA_BA | 2.18 | 0.98 | 1.73 | 0.50 |
| Clinical response rate | CM_BA BA | 2.35 | 3.96 | 3.06 | 0.75 |
|  | CP BA | 0.37 | 1.30 | 0.63 | 0.06 |
|  | CP_BA BA | 3.30 | 2.82 | 2.51 | 0.84 |
|  | FN BA | 3.98 | 4.59 | 4.03 | 0.91 |
|  | SA_BA BA | 4.84 | 0.57 | 1.41 | 0.03 |
|  | CM_FN CM | 1.94 | 0.58 | 1.45 | 0.56 |
|  | EA CM | 0.04 | 0.09 | 0.06 | 0.57 |
|  | FN CM | 0.84 | 0.35 | 0.65 | 0.60 |
|  | CM_FN CM_BA | 2.56 | 4.64 | 2.89 | 0.71 |
|  | FN CM_FN | 0.41 | 0.60 | 0.45 | 0.81 |
|  | CP_BA CP | 6.55 | 1.43 | 4.02 | 0.004 |
|  | CP_BA_EA CP | 3.09 | 14.00 | 4.22 | 0.15 |
|  | CP_CM_BA CP | 4.25 | 26.34 | 8.61 | 0.01 |
|  | CP_EA CP | 2.04 | 1.58 | 2.03 | 0.78 |
|  | CP_NM CP | 4.43 | 15.46 | 6.74 | 0.18 |
|  | CP_SA_BA CP | 5.34 | 84.6 | 6.63 | 0.02 |
|  | CP_WAN CP | 4.70 | 18.56 | 7.06 | 0.40 |
|  | EA CP | 1.17 | 0.35 | 0.62 | 0.17 |
|  | SA_BA CP | 1.71 | 2.87 | 2.27 | 0.58 |
|  | CP_CM_BA CP_BA | 5.39 | 0.83 | 2.13 | 0.01 |
|  | CP_NM CP_BA | 3.61 | 1.02 | 1.68 | 0.20 |
|  | CP_WAN CP_BA | 4.16 | 1.18 | 1.75 | 0.44 |
|  | CP_EA CP_BA_EA | 0.45 | 0.58 | 0.48 | 0.83 |
|  | EA CP_EA | 0.26 | 0.17 | 0.30 | 0.79 |
|  | SA_BA CP_SA_BA | 0.12 | 2.15 | 0.34 | 0.01 |
| Progression rate to Grade 0 on the Modified Ashworth Scale(MAS) | CP_BA CP | 4.01 | 1.21 | 3.39 | 0.26 |
|  | CP_BA_EA CP | 2.30 | 0.76 | 1.83 | 0.47 |
|  | CP_CM_BA CP | 0.42 | 10.23 | 1.86 | 0.03 |
|  | CP_BA_EA CP_BA | 0.22 | 0.72 | 0.54 | 0.44 |
|  | CP_CM_BA CP_BA | 2.21 | 0.11 | 0.55 | 0.045 |
| Spasm relief effective rate | CP_BA CP | 4.01 | 1.21 | 3.39 | 0.26 |
|  | CP_BA_EA CP | 2.30 | 0.76 | 1.83 | 0.47 |
|  | CP_CM_BA CP | 0.42 | 10.23 | 1.86 | 0.03 |
|  | CP_BA_EA CP_BA | 0.22 | 0.72 | 0.54 | 0.44 |
|  | CP_CM_BA CP_BA | 2.21 | 0.11 | 0.55 | 0.04 |

**Table S3** Confidence in network meta-analysis.

| Outcome | Comparison | Within-study bias | Reporting bias | Indirectness | Imprecision | Heterogeneity | Incoherence | Overall confidence |
| --- | --- | --- | --- | --- | --- | --- | --- | --- |
|  |  |  |  |  |  |  |  |  |
| ADL (>1 Month) | CP_CM_BA:SH | Some concerns | Low risk | No concerns | No concerns | No concerns | Major concerns | Low |
|  | CP_NM:SH | No concerns | Low risk | No concerns | No concerns | No concerns | Major concerns | Low |
| ADL (≤1 Month) | CP:CP_SA_BA | Some concerns | Low risk | No concerns | No concerns | No concerns | No concerns | Moderate |
|  | BA:CP | Some concerns | Low risk | No concerns | Some concerns | No concerns | No concerns | Moderate |
| MF (>1 Month) | CP_BA_EA:SA_BA | Some concerns | Low risk | No concerns | No concerns | Some concerns | Major concerns | Low |
|  | CP_BA:CP_BA_EA | No concerns | Low risk | No concerns | Major concerns | No concerns | Major concerns | Very low |
| MF (≤1 Month) | CP:CP_SA_BA | Some concerns | Low risk | No concerns | No concerns | No concerns | No concerns | Moderate |
|  | BA:CP_SA_BA | Some concerns | Low risk | No concerns | No concerns | No concerns | Major concerns | Low |
|  | CP_SA_BA:SA_BA | Some concerns | Low risk | No concerns | No concerns | No concerns | Major concerns | Low |
| Muscle Cramp Severity (>1 Month) | CP:CP_SA_BA | Some concerns | Low risk | No concerns | Some concerns | No concerns | Some concerns | Low |
|  | CP_SA_BA:SA_BA | Some concerns | Low risk | No concerns | Major concerns | No concerns | Some concerns | Low |
|  | BA:CP_SA_BA | No concerns | Low risk | No concerns | Major concerns | No concerns | Some concerns | Low |
| Muscle Cramp Severity(≤1 Month) | CP:CP_SA_BA | Some concerns | Low risk | No concerns | No concerns | Some concerns | Some concerns | Low |
|  | BA:CP_SA_BA | No concerns | Low risk | No concerns | No concerns | Some concerns | Major concerns | Low |
|  | CP_SA_BA:SA_BA | Some concerns | Low risk | No concerns | Major concerns | No concerns | Some concerns | Low |
| Clinical Response Rate (CRR) | CM_FN:EA | No concerns | Low risk | No concerns | No concerns | No concerns | No concerns | High |
|  | CM:CM_EA | Some concerns | Low risk | No concerns | Major concerns | No concerns | No concerns | Low |
|  | CP_SA_BA:EA | Some concerns | Low risk | No concerns | No concerns | No concerns | No concerns | Moderate |
|  | CM_FN:CP | No concerns | Low risk | No concerns | No concerns | No concerns | No concerns | High |
|  | CM_EA:CP | Some concerns | Low risk | No concerns | No concerns | No concerns | No concerns | Moderate |
|  | CM:EA | Some concerns | Low risk | No concerns | No concerns | No concerns | No concerns | Moderate |
| Progression Rate to Grade 0 on the MAS | CP:CP_SA_BA | Some concerns | Low risk | No concerns | No concerns | No concerns | No concerns | Moderate |
|  | BA:FN | Some concerns | Low risk | No concerns | No concerns | No concerns | No concerns | Moderate |
|  | CP_SA_BA:FN | Some concerns | Low risk | No concerns | Major concerns | No concerns | No concerns | Low |
| Spasm Relief Effectiveness Rate | BA:SH | No concerns | Low risk | No concerns | No concerns | Some concerns | No concerns | Moderate |
|  | BA:SA | No concerns | Low risk | No concerns | Some concerns | No concerns | No concerns | Moderate |
